# Sex-specific impact of patterns of imageable tumor growth on survival of primary glioblastoma patients

**DOI:** 10.1186/s12885-020-06816-2

**Published:** 2020-05-19

**Authors:** Paula Whitmire, Cassandra R. Rickertsen, Andrea Hawkins-Daarud, Eduardo Carrasco, Julia Lorence, Gustavo De Leon, Lee Curtin, Spencer Bayless, Kamala Clark-Swanson, Noah C. Peeri, Christina Corpuz, Christine Paula Lewis-de los Angeles, Bernard R. Bendok, Luis Gonzalez-Cuyar, Sujay Vora, Maciej M. Mrugala, Leland S. Hu, Lei Wang, Alyx Porter, Priya Kumthekar, Sandra K. Johnston, Kathleen M. Egan, Robert Gatenby, Peter Canoll, Joshua B. Rubin, Kristin R. Swanson

**Affiliations:** 1 Precision Neurotherapeutics Innovation Program, Mayo Clinic, 5777 East Mayo Blvd , SSB 02-700, Phoenix, AZ 85054 USA; 2 School of Life Sciences, Arizona State University, Tempe, AZ USA; 3 Centre for Mathematical Medicine and Biology, University of Nottingham, Nottingham, UK; 4 Cancer Epidemiology Program, Moffitt Cancer Center, Tampa, FL USA; 5 Department of Neurology, Columbia University Medical Center, New York, NY USA; 6 Northwestern University Interdepartmental Neuroscience Program, Northwestern University Feinberg School of Medicine, Chicago, IL USA; 7 Department of Neurologic Surgery, Mayo Clinic, Phoenix, AZ USA; 8 Department of Pathology, Division of Neuropathology, University of Washington, Seattle, WA USA; 9 Department of Radiation Oncology, Mayo Clinic, Phoenix, AZ USA; 10 Department of Neurology, Mayo Clinic, Phoenix, AZ USA; 11 Department of Radiology, Mayo Clinic, Phoenix, AZ USA; 12 Department of Radiology, Feinberg School of Medicine, Northwestern University, Chicago, IL USA; 13 Department of Neurology, Robert H Lurie Comprehensive Cancer Center, Northwestern University Feinberg School of Medicine, Chicago, IL USA; 14 Department of Radiology, University of Washington, Seattle, WA USA; 15 Cancer Biology and Evolution Program, Moffitt Cancer Center, Tampa, FL USA; 16 Division of Neuropathology, Department of Pathology and Cell Biology, Columbia University Medical Center, New York, NY USA; 17 Department of Pediatrics, Washington University School of Medicine, St Louis, MO USA

**Keywords:** Glioblastoma, Neuroimaging, Sex differences, Biomathematical models

## Abstract

**Background:**

Sex is recognized as a significant determinant of outcome among glioblastoma patients, but the relative prognostic importance of glioblastoma features has not been thoroughly explored for sex differences.

**Methods:**

Combining multi-modal MR images, biomathematical models, and patient clinical information, this investigation assesses which pretreatment variables have a sex-specific impact on the survival of glioblastoma patients (299 males and 195 females).

**Results:**

Among males, tumor (T1Gd) radius was a predictor of overall survival (HR = 1.027, *p* = 0.044). Among females, higher tumor cell net invasion rate was a significant detriment to overall survival (HR = 1.011, *p* < 0.001). Female extreme survivors had significantly smaller tumors (T1Gd) (*p* = 0.010 t-test), but tumor size was not correlated with female overall survival (*p* = 0.955 CPH). Both male and female extreme survivors had significantly lower tumor cell net proliferation rates than other patients (M *p* = 0.004, F *p* = 0.001, t-test).

**Conclusion:**

Despite similar distributions of the MR imaging parameters between males and females, there was a sex-specific difference in how these parameters related to outcomes.

## Background

Glioblastoma (GBM) is the most common primary malignant brain tumor, with a median overall survival of 9 to 15 months [1–3]. According to Ostrom et al. [4], only 35% of patients survive more than 1 year and 4.7% of patients survive more than 5 years after diagnosis. Factors such as age at diagnosis, Karnofsky performance score (KPS), extent of surgical resection, and tumor location have been found to play a significant role in determining the duration of patient survival [5–7], but there is still limited insight into which underlying biological features contribute to a patient becoming a “survival outlier.” To date, there is minimal research on the utility of using pretreatment (pre-tx), image-based volumetric and kinetic variables to identify potential extreme and short-term survivors. Additionally, while it has been consistently identified that GBM incidence is higher among males [8–12] and females GBM patients have better outcomes [8, 12–14], little to no research has focused on sex-specific predictors of survival. The ability to pinpoint relevant predictors of the duration of overall survival has clinical value and identifies areas for future research. By using variables derived from patient clinical information and routinely-obtained, non-invasive MR images, we can establish predictors of survival duration that can be readily assessed in a pre-tx setting. Knowing whether these factors affect males and females in the same way will contribute to guiding research efforts towards best-practice, individualized patient care.

The purpose of this study was to determine whether there are sex-specific predictors of survival outcomes among glioblastoma patients. Using patient data from our multi-institutional brain tumor repository, we tested the significance of eight pre-tx volumetric, kinetic, and clinical variables in predicting extreme and short-term survival. We also tested whether these variables and additional categorical variables, including tumor laterality, extent of resection (EOR), isocitrate dehydrogenase 1 (IDH1) mutation status, and O(6)-methylguanine-DNA methyltransferase promoter (MGMT) methylation status, significantly impacted the overall survival of male and female patients. Throughout the analysis, males and females were tested separately as distinct population groups and their results were compared, allowing us to identify sex-specific impactors of survival outcome among GBM patients.

## Methods

### Imaging

As described in Swanson et al. [15], tumor volumes were segmented from MR images [gadolinium-enhanced T1-weighted (T1Gd), T2-weighted (T2), and T2 fluid-attenuated inversion recovery (T2-FLAIR)] by trained individuals using our in-house thresholding-based software. These volumes were converted to their spherically-equivalent radii for further analysis.

### Biomathematical models and patient-specific tumor kinetics

**Fig. 1:**
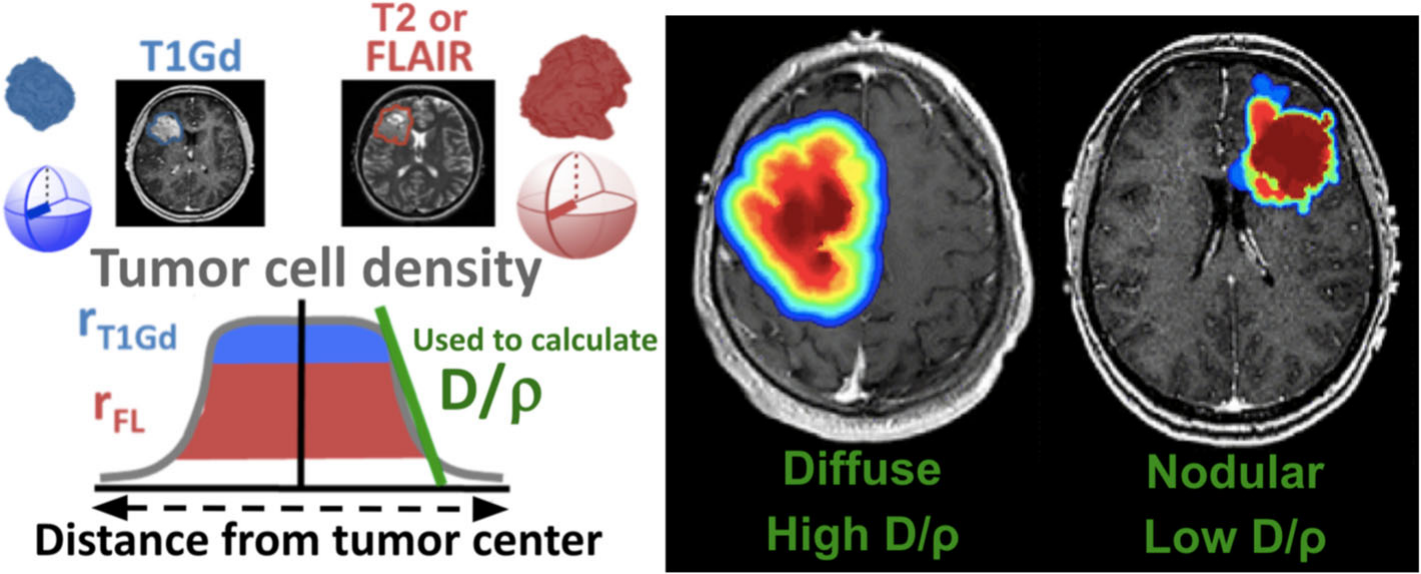
Schematic of determination and interpretation of patient-specific tumor kinetic parameters. Left: After tumors are segmented on T1Gd and T2/FLAIR images, the volumes of the imaging abnormalities are used to calculate the spherically-equivalent tumor radii. By assuming the volume seen on T1Gd corresponds to a high cell density and that on T2/FLAIR to a lower cell density, the relative sizes of the abnormalities on these two imaging modalities gives an estimated profile or slope of the tumor cell density. The ratio of our biomathematical model parameters D/ϱ is a way to quantify this profile. Right: A tumor that has relatively more diffuse invasion compared to tumor cell proliferation (high D/ ϱ) is expected to have a more diffuse distribution of cell density. Conversely, a tumor with relatively more cell proliferation than diffuse invasion (low D/ϱ) is expected to have a more nodular distribution of cell density (red = high tumor cell density, blue = low tumor cell density). Adapted from Baldock et al. 2014 [17] with permission from Oxford University Press (right) and Corwin et al. 2013 [19] (left)

An extensive literature has been generated over the last two decades applying a biomathematical model to simulate patient-specific glioblastoma growth [15–18]. The primary model is referred to as the Proliferation-Invasion (PI) model and is based on two key parameters: the net rate of proliferation, ϱ, and the net rate of invasion, D (Fig. 1). These estimates have been shown to be prognostic of benefit from resection [18], survival [16], and radiation efficacy [20] and can be used to examine therapeutic response [21, 22]. Traditional methods of calculating PI D and ϱ require two pre-tx time points of imaging and these are not always available. We have thus leveraged a second model, the Proliferation-Invasion-Hypoxic-Necrotic-Angiogenesis (PIHNA) model [23], which incorporates necrosis to estimate D and ϱ using one image time point. For more detail, refer to supplement 16 and 17.

### Patient population

Our research lab has amassed a large multi-institutional repository consisting of the clinical patient data and serial, multi-modal MR images of over 1400 glioblastoma patients. From this repository, we identified all newly-diagnosed glioblastoma patients with necessary clinical information (sex, age, and overall survival) and a calculated pre-tx (prior to biopsy or resection) tumor volume from a T1Gd MRI. This cohort was comprised of 494 primary GBM patients (299 males and 195 females). Since the calculation of PIHNA D, PIHNA ϱ, and PI D/ϱ requires both T1Gd and T2 or T2-FLAIR (T2/FLAIR) images, a sub-cohort of patients with sufficient imaging was created from the main cohort in order to study the effect of these variables on survival (223 males and 141 females).

We defined extreme survivors (EXS) as those with overall survival (OS) of 5 years (1825 days) or longer. EXS typically make up less than 5% of glioblastoma patients [4]. However, due to the data collection efforts of a multicenter collaboration researching extreme survival among GBM patients (ENDURES), about 9.5% of patients in this cohort were EXS. When the EXS patients were added to the repository, their medical records were reviewed to confirm the diagnosis of GBM. EXS were compared to Non-EXS (OS< 1825 days). We also compared short-term survivors (STS) (OS≤210 days) [24] and Non-STS (OS> 210 days). The breakdown of the main cohort and the sub-cohort by sex and survival group is shown in Table 1.

**Table 1:** Breakdown of the main cohort and sub-cohort by sex and survival group. Percentages indicate the distribution of males and females in each survival group

	Volumetric and Clinical Data (Main cohort) ***N*** = 494		PI and PIHNA (Sub cohort) ***N*** = 364	
	Male	Female	Male	Female
**All Patients**	299 (60.5%)	195 (39.5%)	223 (61.2%)	141 (38.7%)
**Extreme (OS > 1825 days)**	30 (63.8%)	17 (36.2%)	26 (70.3%)	11 (29.7%)
**Short term (OS < 210 days)**	46 (52.3%)	42 (47.7%)	32 (50%)	32 (50%)

### Statistical analysis

Table 2 outlines the eight quantitative volumetric, kinetic, and clinical variables that were explored in our investigation. Two-sided Student’s t-tests with Welch’s corrections were used to test whether there were significant differences in the eight quantitative variables between the survival groups. Two-sided Cox-Proportional Hazards models (CPH) were used to assess which of the quantitative variables were significant predictors of OS. Parameters that were significant or almost significant (*p* < 0.10) in univariate analysis were compared in multivariate analysis. Kaplan-Meier survival analysis (two-sided log-rank tests) and CPH models were used to assess the impact of the categorical variables on survival. The following categorical variables were included: IDH1 mutation status, MGMT methylation status, tumor laterality, and EOR. T-tests and Kaplan-Meier survival curves were generated using Prism [25] and the CPH models were generated using R studio [26]. All statistical analyses were performed separately for the male and female populations. There was no significant difference in the distribution or mean values of these variables between males and females (Supplement 11).

**Table 2:** Definitions and distributions of the eight quantitative volumetric, kinetic, and clinical variables used in this investigation

Variable used for Investigation	Definition	Male			Female		
		Mean	Median	Range	Mean	Median	Range
Age (years)	Age of patient on date of diagnosis	57.58	58	12–95	58.41	60.5	9–96
T1Gd Radius (mm)	Combined volume of the central non-enhancing necrotic region and surrounding enhanced region of tumor in a pre-tx T1Gd MR image (converted to a spherically- equivalent radius)	19.52	20.10	3.04–33.61	19.27	18.99	4.61–35.08
Necrosis Radius (mm)	Volume of non-enhancing central necrotic region in a pre-tx T1Gd MR image (converted to a spherically- equivalent radius)	11.39	11.69	0.00–26.54	11.37	11.33	0.00–27.06
Contrast- enhancing (CE) thickness (mm)	Average linear thickness of the contrast-enhancing region in a pre-tx T1Gd MR image (calculated as the difference between the T1Gd radius and the necrosis radius)	8.16	7.85	2.55–18.94	7.89	7.59	0.32–23.26
T2 /FLAIR radius (mm)	Volume of the pre-tx T2 or T2-FLAIR MR image (converted to a spherically- equivalent radius)	27.11	28.31	9.94–39.55	26.98	27.86	9.99–42.81
PIHNA D (mm^2^/year)	Net tumor cell diffuse invasion rate	32.34	28.99	1.45–145.3	36.25	23.03	0.37–289.9
PIHNA ϱ (year ^−1^)	Net tumor cell proliferation rate	65.88	18.25	1.83–1825	82.40	18.25	1.83–1825
PI D/ϱ (mm^2^)	Relative tumor invasiveness	2.19	1.65	0.0034–10.26	2.12	1.28	0.0034–10.70

### Decision trees

The decision trees (DT) in this study were created using R [26], accompanied by a package called *rpart* [27], which allows effective decision tree pruning. Six DT were produced in total, grouped into 3 pairs. Within each pair, one tree was created using the male population and the other was created using the female population. The PI and PIHNA subcohort of patients (223 males and 141 females) was used to create the training (70% of population) and testing (30%) groups and 10-fold cross validation was used to ensure the generalizability of the results. For each tree, accuracy and sensitivity (EXS and STS are considered condition positive) are reported for the training group, testing group, and the full cohort (training + testing). All six trees were constructed using the eight quantitative pre-tx variables: age, T1Gd radius, necrosis radius, CE thickness, T2/FLAIR radius, PIHNA D, PIHNA ϱ, and PI D/ϱ.

### Study approval

All featured patients either provided informed consent or were approved for retrospective research before inclusion in this investigation. All methods were carried out in accordance with the relevant guidelines and regulations. All experimental protocols, including the usage and collection of patient data, were carried out under Mayo Clinic institutional review board approvals.

## Results

### Variables associated with extreme and short-term survival

Student’s t-tests were performed separately on males and females and compared the following groups: EXS vs Non-EXS, EXS vs STS, and STS vs Non-STS. The results of this analysis can be found in Table 3**.** When compared to the rest of the male population, EXS were significantly younger (*p* = 0.005) and STS were significantly older (*p* < 0.001). Male EXS had significantly smaller ϱ when compared to male Non-EXS (*p* = 0.004). When compared to the rest of the female population, female EXS were significantly younger (*p* = 0.032) while female STS were significantly older (p < 0.001). Female EXS had significantly smaller T1Gd radii compared to female Non-EXS (*p* = 0.010). Compared to the rest of the female population, female EXS had significantly smaller D (*p* = 0.008) and female STS had significantly larger D (*p* = 0.018). Female EXS had significantly smaller ϱ compared to female Non-EXS (*p* = 0.001).

**Table 3:**
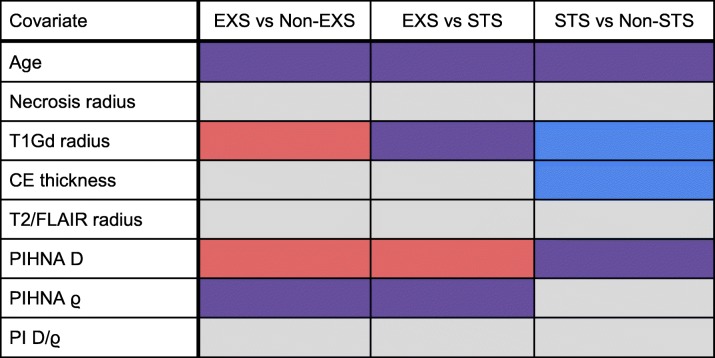
Results of the t-test comparisons of the eight quantitative volumetric and clinical variables between the survival groups for males and females. Purple boxes indicate that the means of the variables were significantly different between the survival groups within both the male and female populations. Red boxes indicate a significant difference within the female population and blue indicate a significant difference within the male population. Gray boxes indicate that neither population showed a significant difference in the means of the variables between the survival groups. Detailed results of t-tests can be found in Supplement 13

**Fig. 2:**
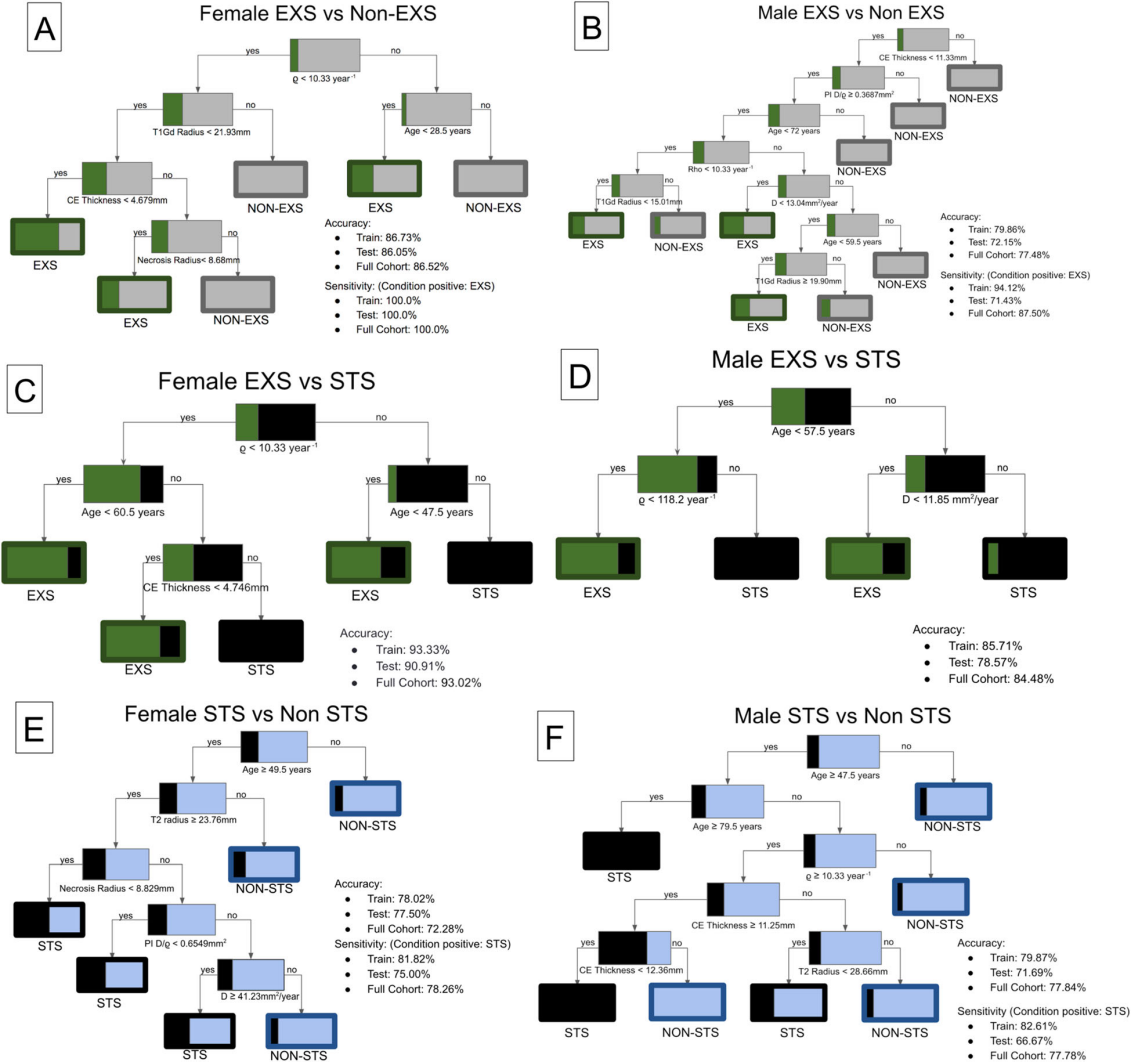
Decision trees binning male and female EXS, Non-EXS, STS, and Non-STS based on patient and tumor characteristics. At each node, color (green for EXS, gray for Non-EXS, black for STS, and blue for Non-STS) and percentages indicate concentration of each group. **a** Female EXS vs Non-EXS DT (*n* = 141). **b** Male EXS vs Non-EXS DT (*n* = 223). **c** Female EXS vs STS DT (*n* = 43). **d** Male EXS vs STS (*n* = 58). **e** Female STS vs Non-STS DT (n = 141). **f** Male STS vs Non-STS DT (n = 223)

In the female EXS vs Non-EXS DT **(**Fig. 2a and b**)**, the nodes that predicted EXS with 100% sensitivity included T1Gd radius < 21.93 mm and age < 28.5 years. Notably, all male EXS had CE thickness shorter than 11.33 mm, PI D/ϱ above 0.3687 mm^2^, and age below 72 years. In the female EXS vs STS DT **(**Fig. 2c and d**)**, the nodes that best predicted female EXS included ϱ < 10.33 year ^− 1^ and CE thickness < 4.746 mm and the node that best predicted female STS was age ≥ 47.5 years. In the male DT, the node that best predicted EXS was ϱ < 118.2 year ^− 1^ and the node that best predicted STS was D ≥ 11.85 mm^2^/year. The third pair of DT sorted males and females into STS and Non-STS groups **(**Fig. 2e and f**)**. Among females, the nodes that best predicted STS included age ≥ 49.5 years, T2/FLAIR radius ≥ 23.76 mm, and D ≥ 41.23 mm^2^/year. In the male DT, the nodes that most accurately predicted STS included age ≥ 47.5 years, ϱ ≥ 10.33 year ^− 1^, and CE thickness between 11.25 mm and 12.36 mm.

### Variables associated with overall survival

Univariate and multivariate CPH analyses **(**Table 4**)** were utilized to determine which variables significantly influenced the overall survival of GBM patients. Variables that were significant or almost significant (*p* < 0.10) in univariate analysis were analyzed in multivariate analysis. In the male multivariate CPH, factors found to independently influence survival included: age (HR = 1.030, *p* < 0.001) and T1Gd radius (HR = 1.027, *p* = 0.044). In the female multivariate CPH analysis, age (HR = 1.021, *p* = 0.006) and PIHNA D (HR = 1.011, p < 0.001) were identified as significant independent prognostic factors.

**Table 4:** Results of univariate and multivariate CPH analyses for males and females. Factors that were almost significant (*p* < 0.10) or significant in univariate analysis were included in the multivariate analysis

	Univariate			Multivariate		
Covariate	HR	95% CI	*p*-value	HR	95% CI	*p*-value
**Males**						
Age	1.027	1.018–1.037	**< 0.001**	1.030	1.017–1.044	**< 0.001**
Necrosis radius	1.018	0.996–1.040	0.118			N/A
T1Gd radius	1.024	1.003–1.046	**0.025**	1.027	1.001–1.054	**0.044**
CE Thickness	1.028	0.989–1.068	0.161			N/A
T2/FLAIR radius	0.996	0.972–1.020	0.744			N/A
PIHNA D	1.003	0.997–1.010	0.266			N/A
PIHNA ϱ	1.001	1.000–1.001	0.064	1.000	0.999–1.001	0.637
PI D/ϱ	0.932	0.872–0.996	**0.038**	0.951	0.880–1.029	0.210
**Females**						
Age	1.028	1.015–1.041	**< 0.001**	1.021	1.006–1.037	**0.006**
Necrosis radius	1.017	0.991–1.042	0.204			N/A
T1Gd radius	1.026	1.000–1.052	**0.048**	0.993	0.964–1.023	0.641
CE Thickness	1.037	0.988–1.088	0.143			N/A
T2/FLAIR radius	1.017	0.989–1.045	0.232			N/A
PIHNA D	1.011	1.006–1.016	**< 0.001**	1.011	1.005–1.017	**< 0.001**
PIHNA ϱ	1.001	1.000–1.002	0.052	1.000	0.999–1.002	0.801
PI D/ϱ	0.996	0.937–1.059	0.906			N/A

### IDH1 mutation

Since IDH1 mutation has been previously identified as significant predictor of long-term survival [14], we analyzed the impact of sex and IDH1 status on the overall survival of our patient cohort. Among the 120 patients in the main cohort that had available IDH1 status, there were 69 wild-type (wt) and 8 mutant (mut) male patients and 39 wt and 4 mut female patients. When analyzing the entire population (both males and females), there was a trend towards IDH1 mut patients having better survival (log-rank, *p* = 0.071). Among females, IDH1 mut survived significantly longer than IDH1 wt patients (log-rank, p = 0.008), but among males, the survival difference was not significant (log-rank, *p* = 0.924) (Supplement 1). This analysis is limited by the small cohort of IDH1 mut patients, but it was notable that all 4 IDH1 mut females survived at least 3 years, making them all long-term survivors [28].

### MGMT methylation

Methylation of the MGMT promoter has been found to be more common in long-term survivors [29], so we also assessed the impact of MGMT methylation on the survival of our population cohort. Ninety patients from the main cohort had available MGMT methylation status, which comprised of 32 females (12 methylated and 20 unmethylated) and 58 males (18 methylated and 40 unmethylated). Methylated patients had significantly better survival than unmethylated patients among males (log-rank, *p* = 0.013), females (*p* = 0.007), and the entire population (males and females) (*p* < 0.001) (Supplement 4). Multivariate CPH analyses that assessed the impact of MGMT status on survival while accounting for age showed that MGMT status significantly impacted survival for males (*p* = 0.004) and females (*p* = 0.037). Among EXS with available MGMT methylation status (*n* = 15), 50% (*n* = 5) of males and 60% (*n* = 3) of females had MGMT methylation, while among Non-EXS (*n* = 75), 29% (*n* = 14) of males and 33% (*n* = 9) of females had MGMT methylation, suggesting that MGMT methylation was more common among both male and female EXS.

### Laterality

Using pre-tx T1Gd MR images, we determined the laterality of each patient’s tumor, classifying the tumors as being located in the right hemisphere, left hemisphere, or both hemispheres (bilateral). The impact of tumor laterality on survival was assessed separately for males and females, and the results were compared. Among males, there were 129 left hemisphere GBMs, 154 right hemisphere GBMs, and 11 bilateral GBMs, and among females there were 86 left hemisphere GBMs, 96 right hemisphere GBMs, and 9 bilateral GBMs. Laterality could not be determined for 5 male and 4 female patients.

Male patients with tumors on the left side tended to have better survival than males with tumors on the right side (log-rank, *p* = 0.077) and had significantly better survival than males with bilateral tumors (*p* = 0.010) (Supplement 6). In a multivariate CPH analysis that also accounted for extent of resection, tumor location in the left hemisphere was found to be a significant independent predictor of improved survival outcome for males (*p* = 0.017) (Supplement 14). There were more EXS than STS among males with tumors on the left side and there were almost twice as many STS as EXS among males with tumors on the right side. Laterality did not have a significant impact on survival for female patients (CPH, *p* = 0.299) (Supplement 14). There was no significant difference in survival between females with left and right hemisphere tumors (log-rank, *p* = 0.218), and females with bilaterally located tumors did not have significantly worse survival when compared to females with non-bilateral tumors (bilateral vs left *p* = 0.272, bilateral vs right *p* = 0.471) (Supplement 6).

### Extent of resection

Our investigation evaluated whether the extent of initial surgical intervention, a known prognostic factor among GBM patients, had the same prognostic value for both male and female GBM patients. Patient EOR status, categorized as gross total resection (GTR), subtotal resection (STR), or biopsy, was obtained from the patient records. From the main cohort of 494 patients, 211 males (83 GTR, 83 STR, and 45 biopsy) and 136 females (54 GTR, 55 STR, and 27 biopsy) had available EOR status.

EOR had a significant impact on the survival of male GBM patients. GTR males had significantly better survival than STR males (log-rank, *p* = 0.033) (Supplement 9) and males who received some surgical resection (GTR or STR) had significantly better survival than males who only received a biopsy (*p* = 0.013) (Supplement 8). Cochran-Armitage Trend Test showed that there was significant trend towards male EXS receiving more extensive resections and male STS receiving less extensive resections or biopsies (*p* = 0.027). Female who received resection (GTR or STR) trended towards improved survival compared to biopsy females (log-rank, *p* = 0.077) (Supplement 8), but there was no significant difference in survival between GTR females and STR females (*p* = 0.992) (Supplement 9). Additionally, EOR did not significantly impact female survival in univariate CPH analysis (*p* = 0.180) (Supplement 14). Trend test showed that there was an insignificant trend towards female EXS receiving more extensive resections and female STS receiving less extensive resections or biopsies (*p* = 0.098).

### Patients receiving current standard of care

Due to the timespan over which they were collected, the patients in our cohort received a wide variety of treatment protocols. In order to ensure that our results maintain significance among patients who receive the current standard of care (maximal safe resection followed by concurrent temozolomide and radiation therapy), we created a subset of patients who received this treatment protocol (Stupp protocol patients) [30] and tested which factors were associated with overall survival among those patients (Supplement 15). In this limited subpopulation, we had 113 males and 66 females (Supplement 15A). Among females, PIHNA D was a significant independent predictor of overall survival and among males, PIHNA ϱ was a significant independent predictor of overall survival (Supplement 15B).

## Discussion

**Fig. 3:**
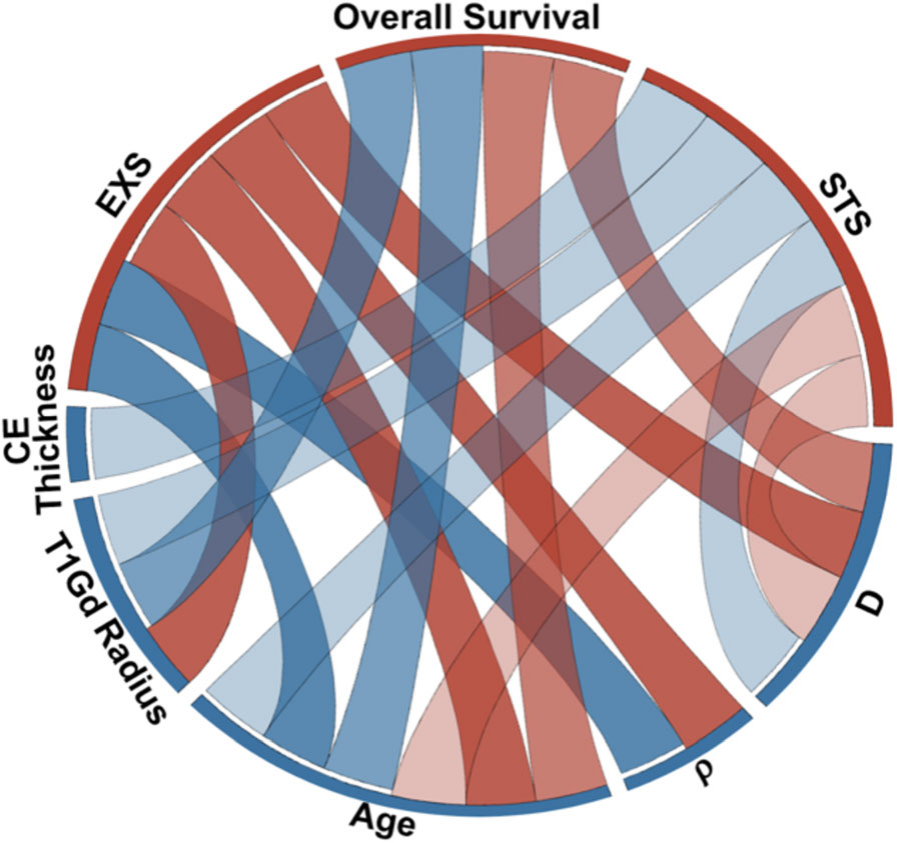
Sex differences in the impact of image-based parameters on survival [31]. The differences between the connections of the red and blue ribbons represent sex differences in the prognostic significance of image-based tumor and patient characteristics. The bottom portion of the outer ring lists the relevant quantitative variables and the top portion shows the three aspects of survival that are associated with these variables (EXS, STS, and Overall Survival). Red ribbons indicate significant relationships for female patients between the parameter and the survival group and blue ribbons indicate significant relationships for male patients. Variables that were significant in multivariate CPH are connected to the Overall Survival segment and variables that were significant in Student t-tests with Welch’s correction are connected to the relevant EXS or STS segments

While there are no differences in the distributions of these quantitative and categorical variables between males and females, this investigation found that there are sex-specific differences in the impact that these variables have on patient survival **(**Fig. 3**)**.

### Impact of quantitative variables on survival

Tumor cell diffuse invasion rate (PIHNA D) is strongly negatively correlated with overall survival for females across the various analyses and is not consistently significant for males. Notably, both when EOR was included in multivariate CPH analysis (Supplement 14) and when only Stupp protocol patients were considered (Supplement 15B), PIHNA D was still an independent predictor of survival for females. Although it was not significant in the CPH multivariate analysis, it is notable that males had a significant positive association between overall survival and PI D/ϱ in univariate analysis **(**Table 4**)**. This suggests that more nodular tumors at time of diagnosis are associated with worse prognosis for males, which is contrary to the finding that more diffusely invasive tumors are associated with worse prognosis for females.

Among males, total tumor size (T1Gd radius) is negatively correlated with overall survival across the statistical analyses **(**Tables 3 and 4**)**. In the DT analyses, CE thickness, a component of total tumor size, is a highly sensitive predictor of survival outcome **(**Fig. 2b and f**)**. While total tumor size is not continuously associated with survival for females in the same way that it is for males, smaller total tumor size (T1Gd radius) is significantly associated with EXS for females. DT analysis showed that nodes isolating females with below average necrosis radii and CE thickness, both components of overall tumor size, were highly sensitive predictors of EXS **(**Fig. 2a and c**)**. When the mean T1Gd radius of EXS was compared to the mean T1Gd radius of other survival groups, the mean radius of EXS was significantly smaller **(**Table 3**)**. Univariate CPH found that T1Gd radius size was a significant predictor of survival for females **(**Table 4**)**, but if EXS were excluded from the analysis, this relationship is no longer significant (*p* = 0.503). These results suggest female extreme survivors have smaller pre-tx T1Gd radii, but T1Gd radius is not negatively correlated with overall survival for females in general.

Age is known to have a significant impact on the survival of glioblastoma patients [5–7] and this analysis confirmed that age significantly impacts the survival of both males and females. Across the analyses, older age at time of diagnosis is consistently associated with shorter survival, while younger age is associated with longer survival **(**Tables 3 and 4**)**.

Lower tumor cell proliferation rates (PIHNA ϱ) are associated with EXS for both males and females. DT analysis and statistical analysis both showed that low proliferation rates were associated with EXS **(**Table 3 and Fig. 2c and d**).** Low tumor cell proliferation rates appear to be predictive of long-term survival for both males and females, but high rates do not appear to predict short-term survival.

### Impact of categorical variables on survival

While Schiffgens et al. [32] found that only IDH1 mutant males demonstrate significantly improved survival compared to IDH1 wild-type males, our investigation found the opposite, that only IDH1 mutant females demonstrate significantly improved survival when compared to their wild-type counterparts (Supplement 1). While our investigation into this matter is limited by a small cohort of IDH1 mutants, our finding is in concurrence with the findings of Yang et al. [33], who grouped females by genetic similarities and found that the longest-living female cohort predominantly consisted of IDH1 mutant females. They did not see this effect for males. The findings of Schiffgens et al. [32] and Yang et al. [33] make a compelling case for the need to consider sex in IDH1-related research.

Previous studies have demonstrated that MGMT promoter methylation is a significant independent prognostic factor [34] and is more common among long-term survivors [29, 35]. Despite having a small sample of patients with known MGMT methylation status, our analysis was able to confirm that, for both males and females, MGMT methylation was more common among extreme survivors and was a significant independent prognostic factor. Previous studies have also found that the survival benefit of MGMT methylation was stronger or only significant among female patients [32, 36], but our analysis did not see any evidence of females benefiting more from MGMT methylation than males.

In this investigation, GBM laterality impacted male survival, but had no impact on female survival. Even after accounting for EOR, males with tumors located in the left hemisphere had a significant survival advantage compared to males with tumors located in the right hemisphere. Ellingson et al. [37] found that patients who responded favorably to chemotherapy, patients with prolonged survival, and patients with specific genetic modifications, like MGMT promoter methylation and IDH1 mutation, had tumors that clustered in areas of the left hemisphere of the brain. Additional research will need to be conducted on the relationship between genetic modifiers, laterality, sex, and survival.

Previous literature has identified extent of resection as a significant predictor of overall survival for GBM patients [6, 18, 38, 39], but whether EOR has the same impact on survival for males and females has not been clearly elucidated. Our analysis found that EOR has a significant impact on the survival of male GBM patients, with a more complete resection being associated with longer survival and potentially extreme survival. Among females, there was a survival benefit associated with receiving resection, but the extent of resection did not have a significant impact on survival. These findings suggest that EOR may have a sex-specific impact on survival, but further study will be required to fully understand the extent of this difference.

### Limitations and further work

Due to the utilization of retrospective clinical data, it was not possible to control for all confounding factors and bias within our dataset. Some of the factors contributing to the heterogeneity of our cohort include the variety of institutions where patients were treated, the span of the years over which the patients were treated, the relatively small subset of patients with testing for molecular markers, the variety of treatment protocols given to our patients, and the inclusion of IDH1 mutated glioblastomas within our cohort [40]. Our utilization of a large cohort of almost 500 patients allows for the mitigation of some of these confounding effects and a sub-analysis of patients with the same treatment protocol largely confirmed the results from the full cohort, but further study is needed to validate the results of this study. Once validated, further basic biological and prospective clinical investigations will be necessary to elucidate the mechanism and clinical implication of these observed differences.

## Conclusion

Taken together, these results emphasize the need to consider sex as a relevant biological factor in all glioblastoma-related research. Sex has been shown to play a significant role in many clinically relevant aspects of GBM, and yet, many studies do not report patient sex and those that do often do not consider sex when analyzing their results. The consideration of the role of sex in tumor behavior, incidence, growth, and treatment response will only lead to higher-quality, more individualized knowledge and care for glioblastoma patients.

## Supplementary information

**Additional file 1.**

## Data Availability

The datasets generated during and/or analyzed during the current study are not publicly available due to institutional review board requirements but are available from the corresponding author on reasonable request.

## Abbreviations

GBM: glioblastoma
KPS: Karnofsky performance score
pre-tx: pre-treatment
EOR: extent of resection
IDH1: isocitrate dehydrogenase 1
MGMT: O(6)-methylguanine-DNA methyltransferase promoter
T1Gd: gadolinium-enhanced T1-weighted MRI
T2: T2-weighted MRI
T2-FLAIR: T2 fluid-attenuated inversion recovery MRI
PI: proliferation-invasion model
PIHNA: Proliferation-Invasion-Hypoxic-Necrotic-Angiogenesis model
T2/FLAIR: T2 or T2-FLAIR MRI
EXS: extreme survivors
STS: short-term survivors
OS: overall survival
ENDURES: Environmental dynamics underlying responsive extreme survivors consortium
CPH: cox proportional hazard model
CE: contrast-enhancing
DT: decision tree
wt: wild-type
mut: mutated
GTR: gross total resection
STR: sub-total resection
