# Supplementary material for: Sex-specific impact of patterns of imageable tumor growth on survival of primary glioblastoma patients
Source: BMC Cancer. 2020 May 19;20:447. doi: 10.1186/s12885-020-06816-2 (PMC7238585; doi:10.1186/s12885-020-06816-2)
Supplement: Supplementary file 1 — Additional file 1. [file 12885_2020_6816_MOESM1_ESM.docx]

**Supplement**

**IDH1 Mutation**

**
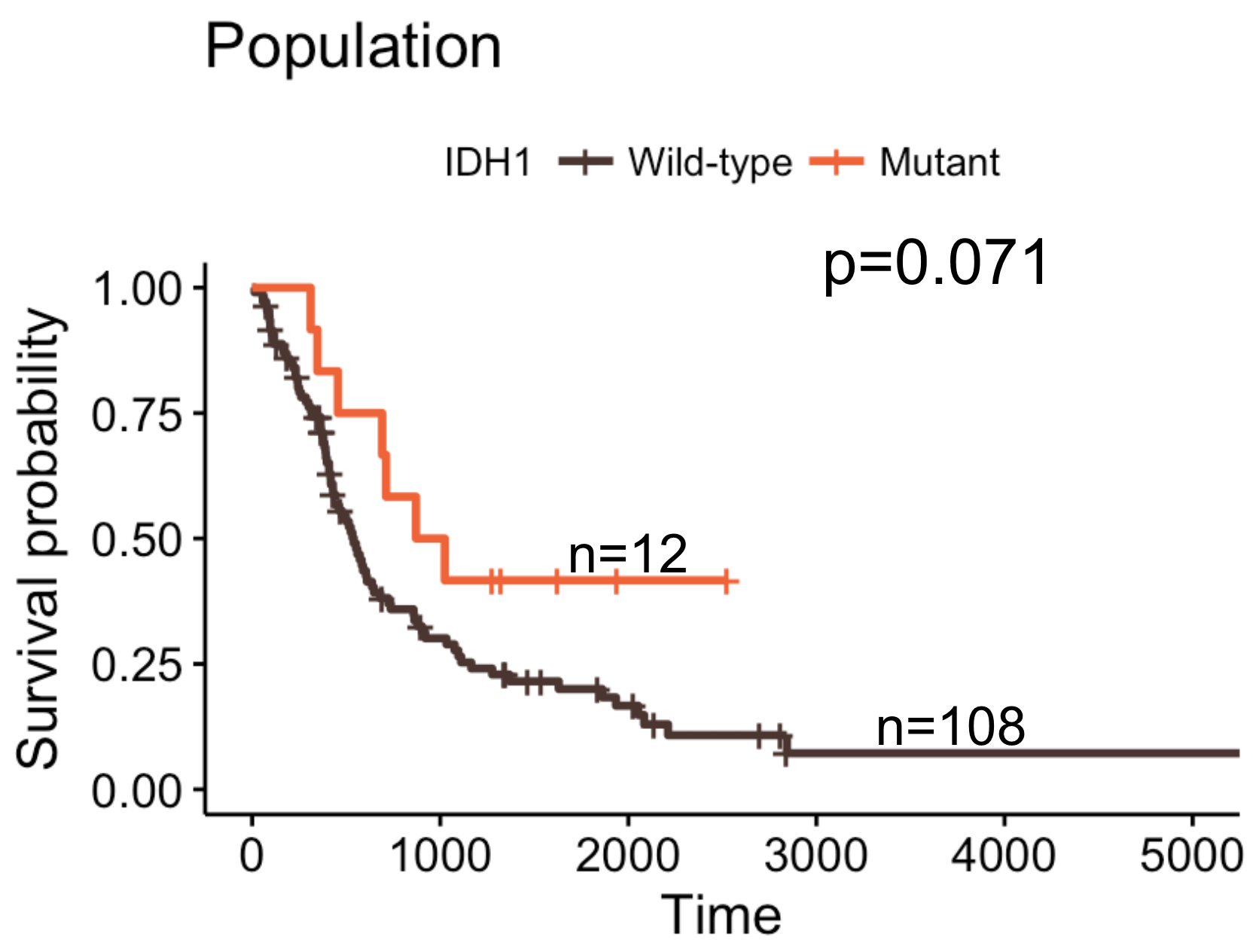

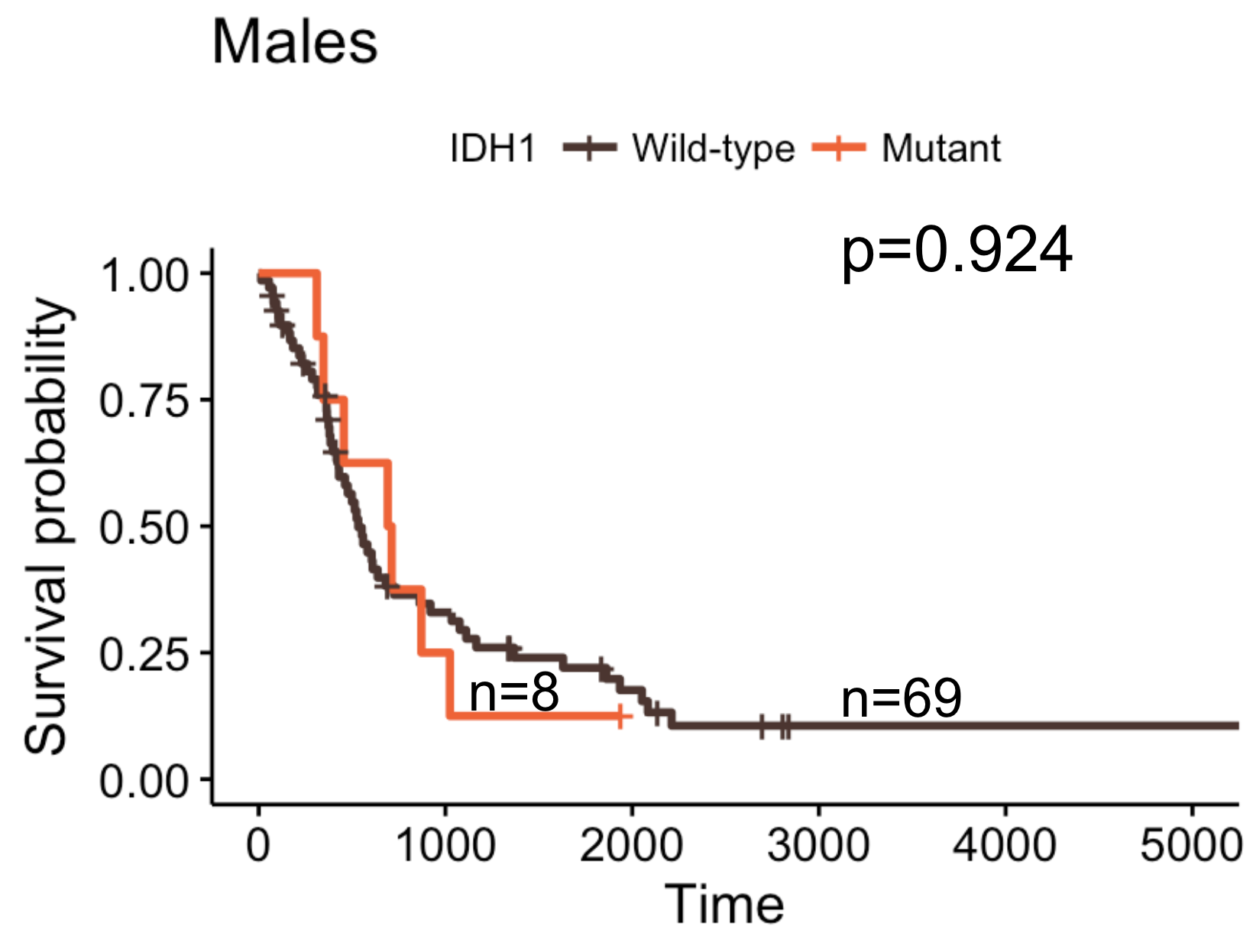
**
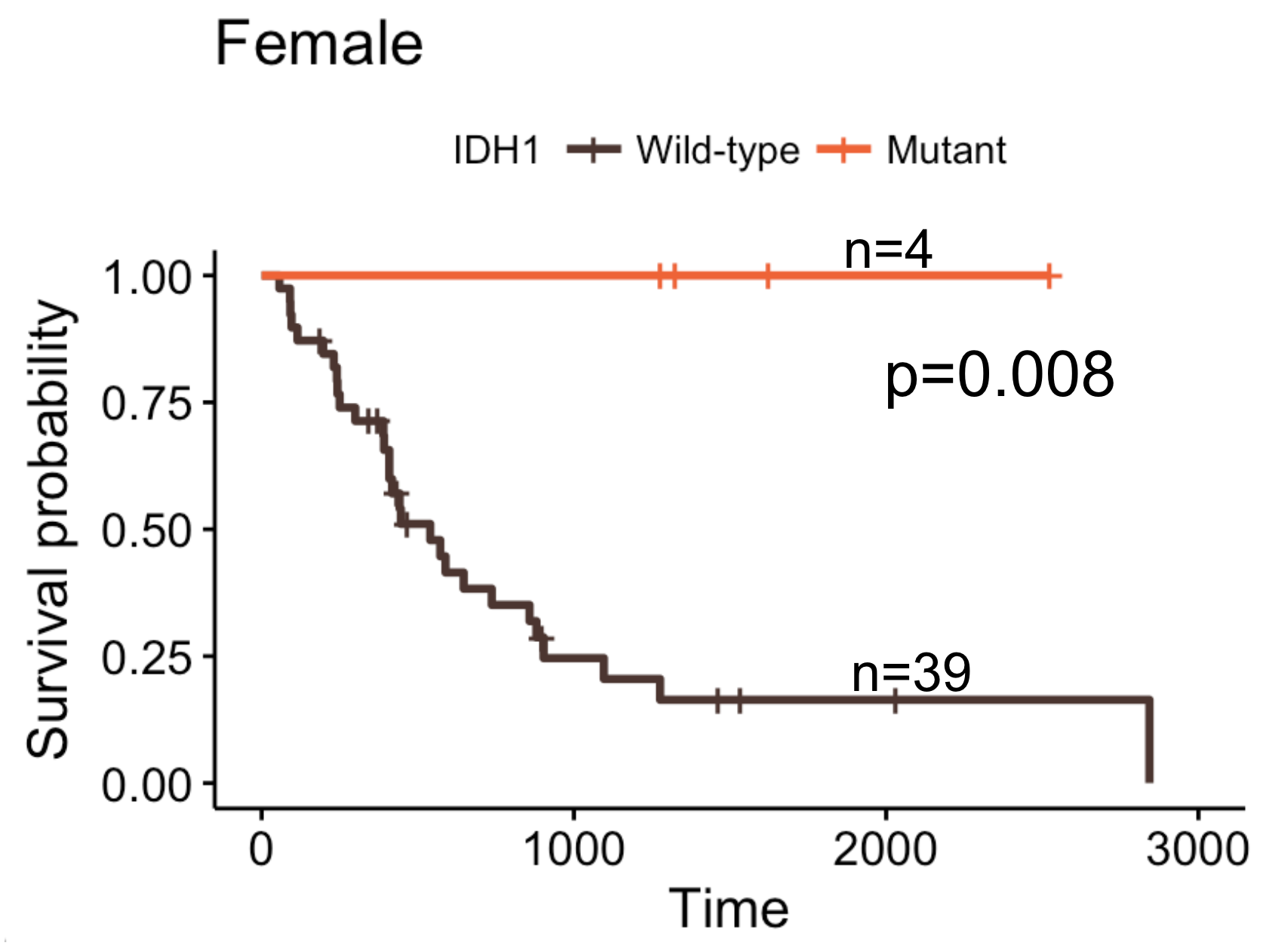


**Supplement 1.** IDH1 mutation only significantly stratifies survival among females.

|  | HR | 95% CI | p-value |
| --- | --- | --- | --- |
| Age | 1.024 | 1.005-1.044 | **0.015** |

**Supplement 2.** Univariate CPH analysis of 108 IDH1 wild-type patients shows that age is a significant predictor of survival


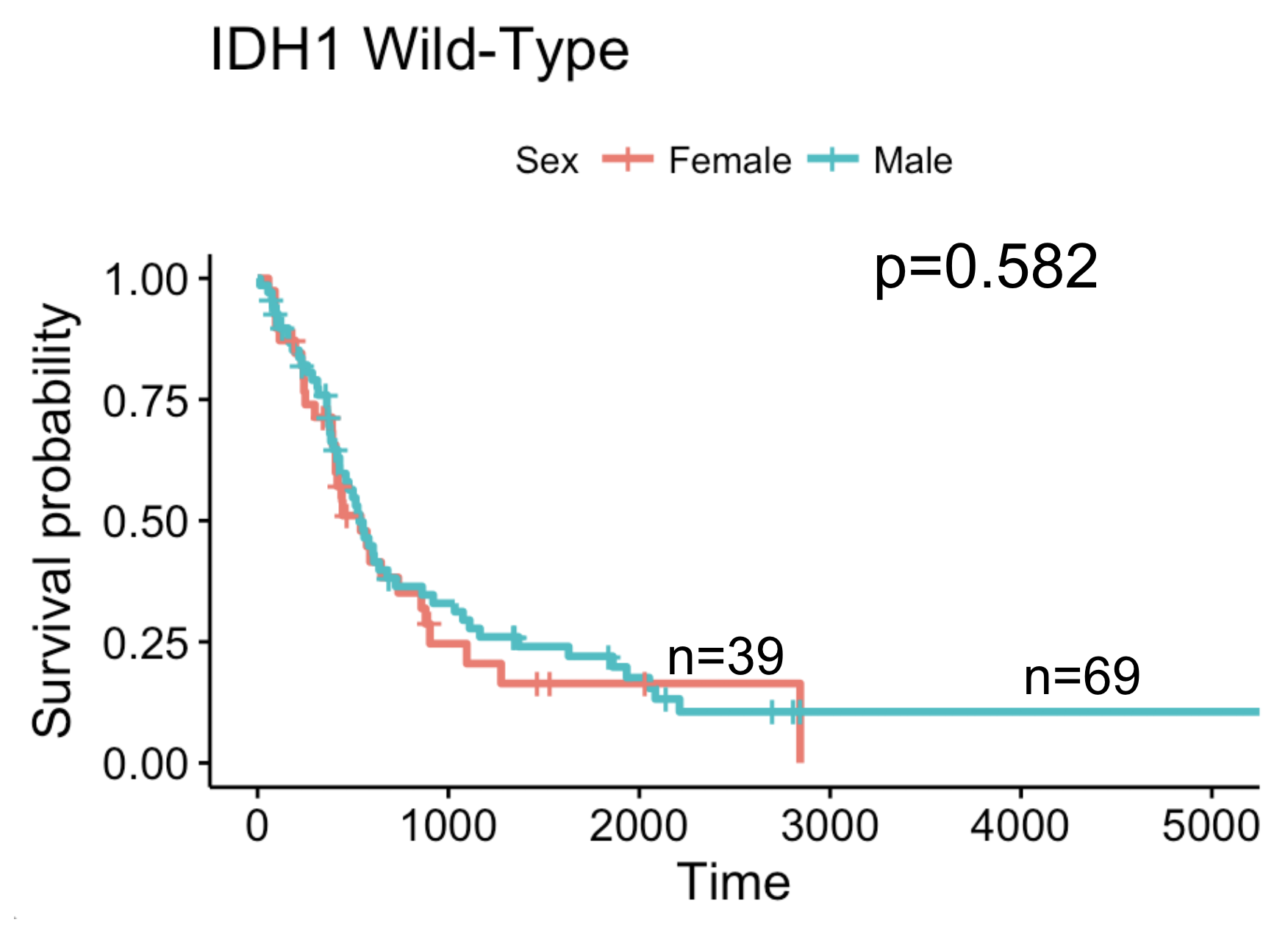

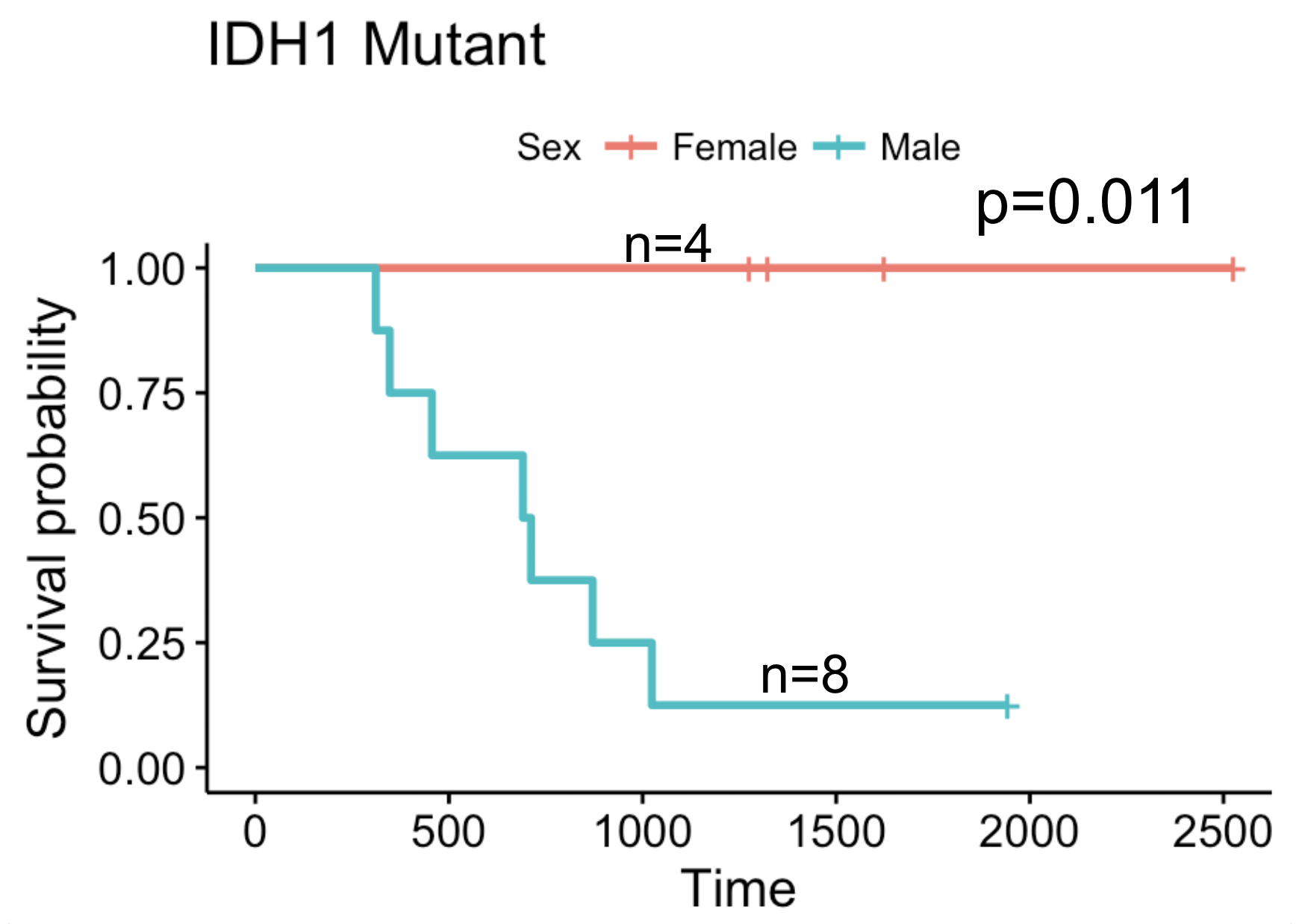


**Supplement 3.** IDH1 mutant females had significantly better survival than IDH1 mutant males.

**MGMT Methylation**


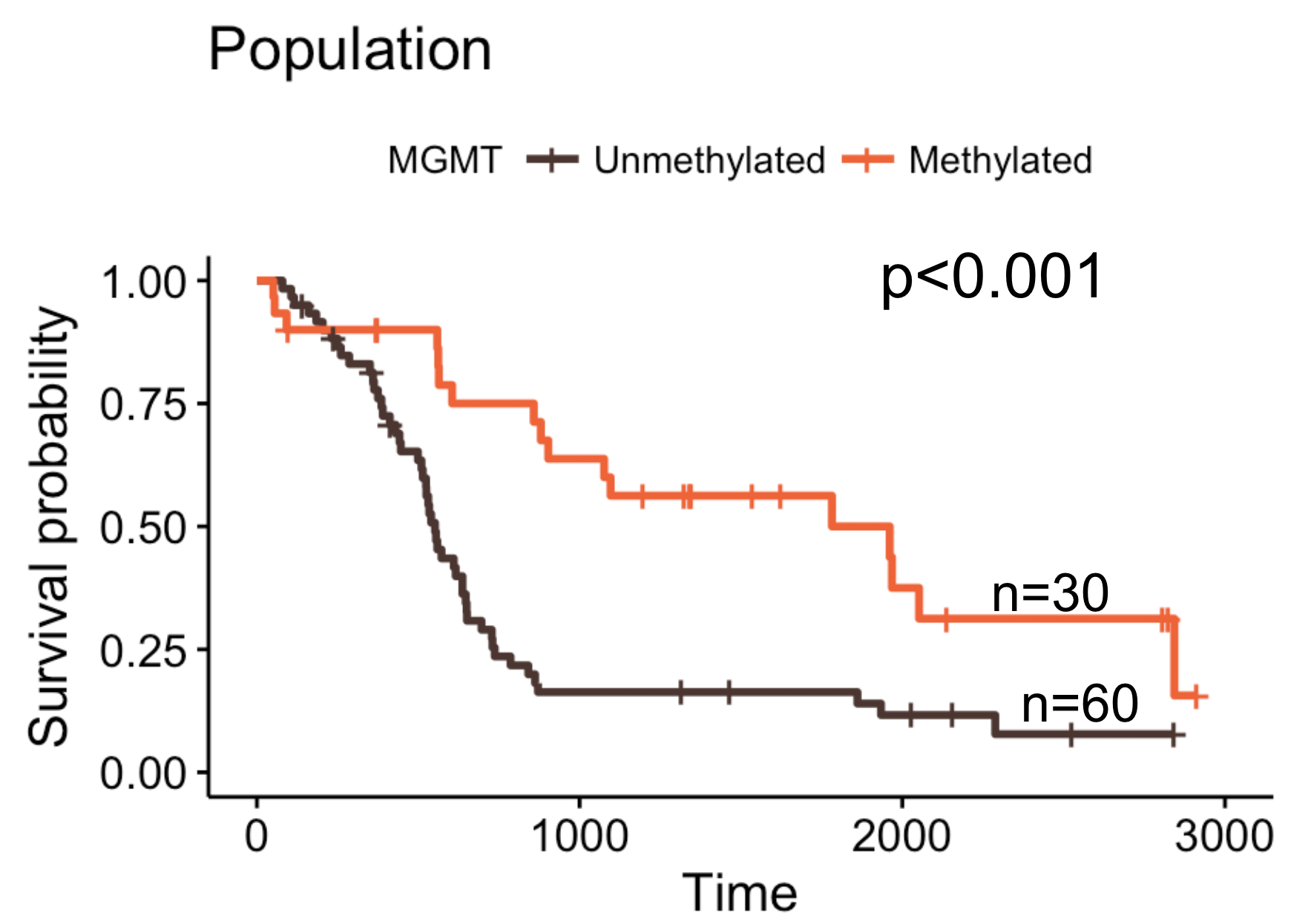

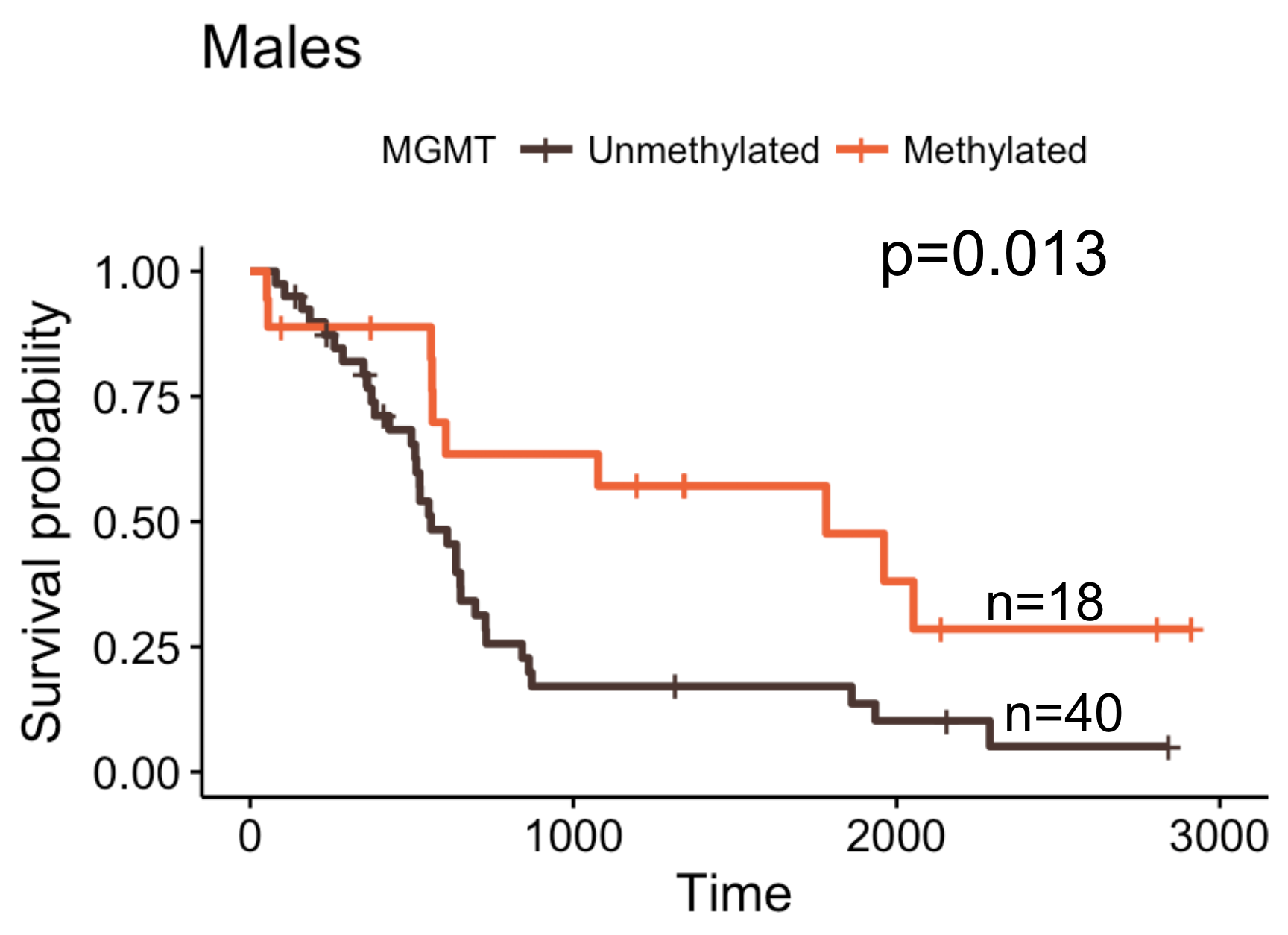

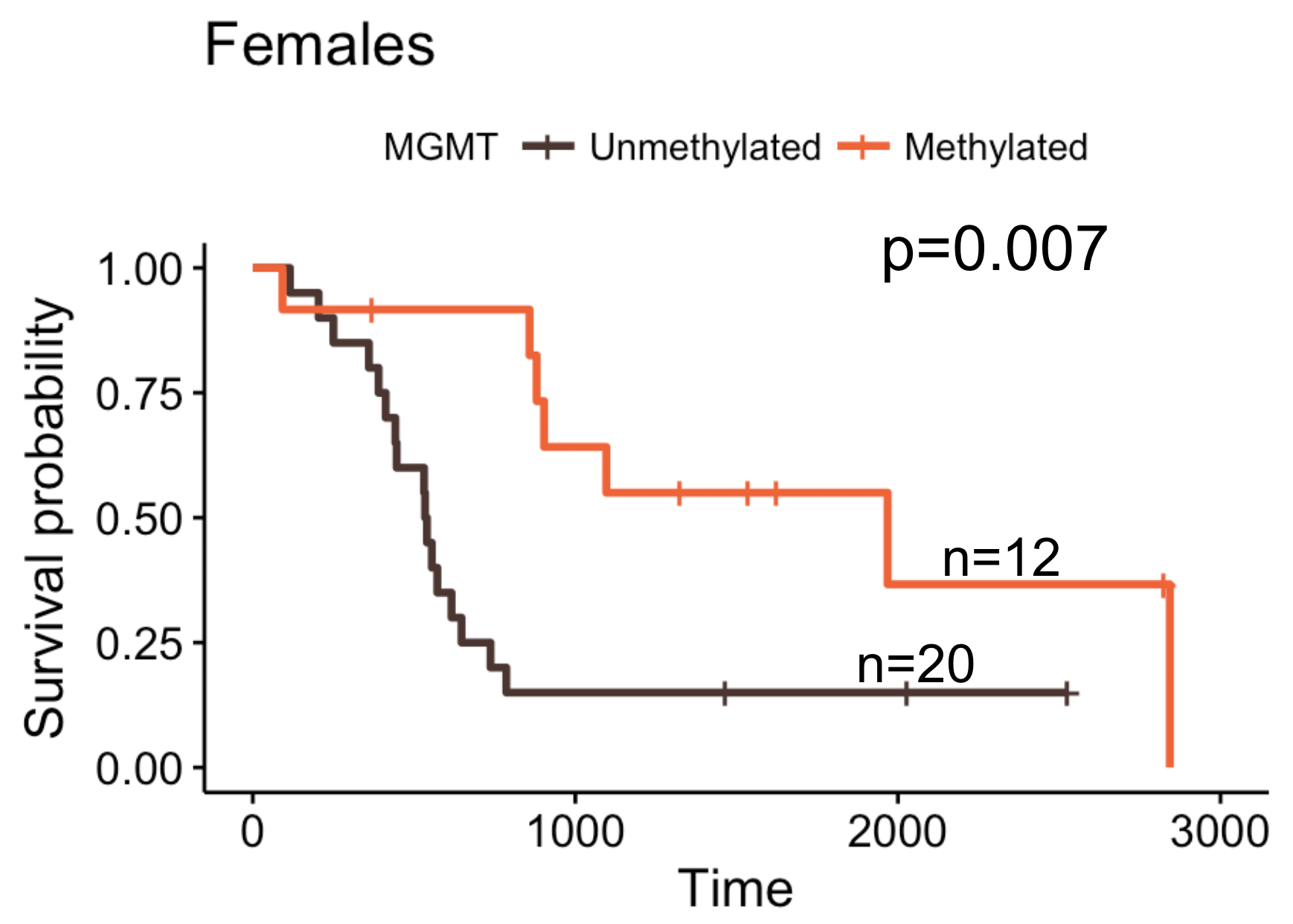


**Supplement 4.** Patients with MGMT methylation have significantly better survival than those with unmethylated MGMT.


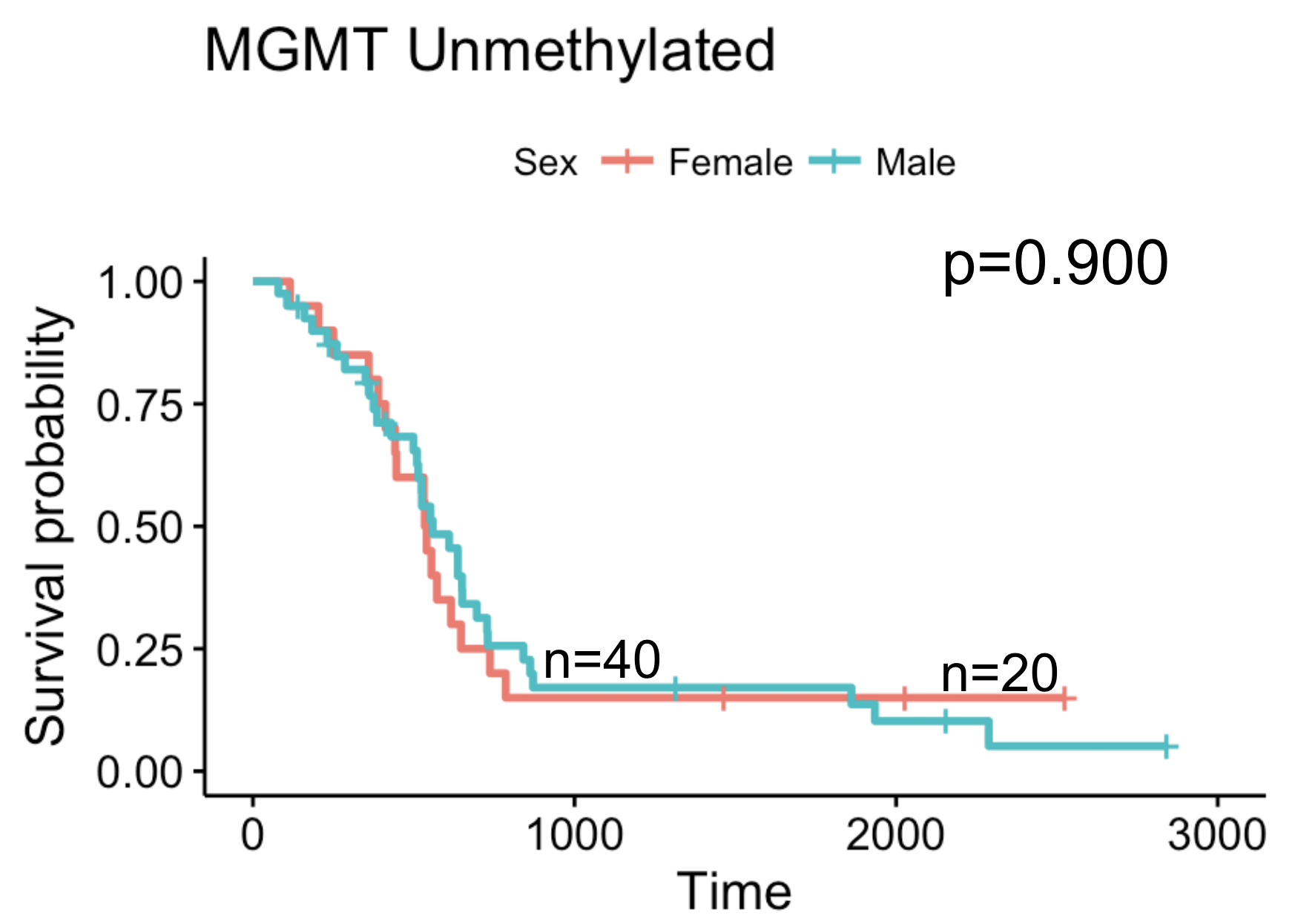

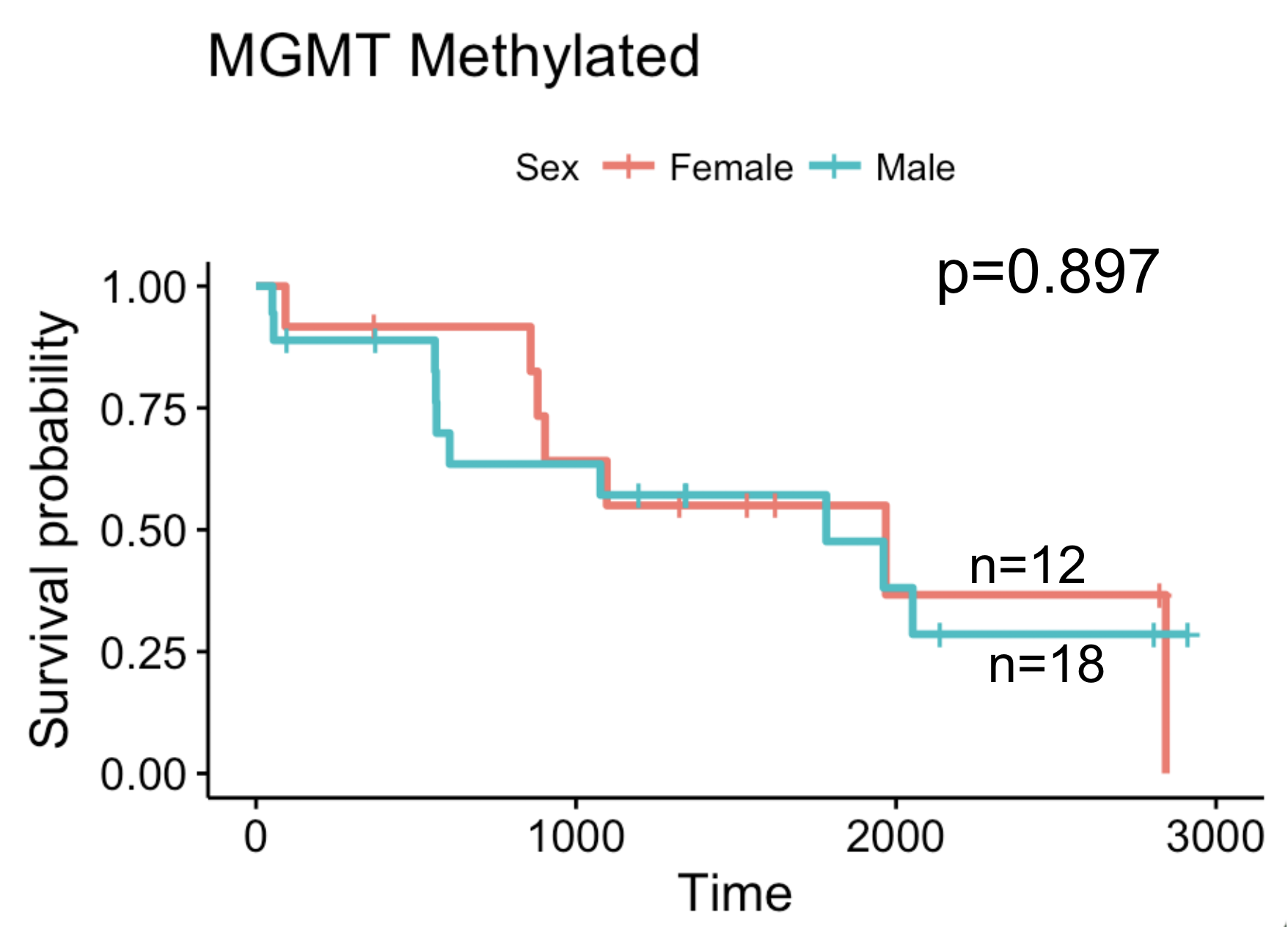


**Supplement 5.** No difference in survival between males and females among MGMT methylated and unmethylated patients.

**Laterality**


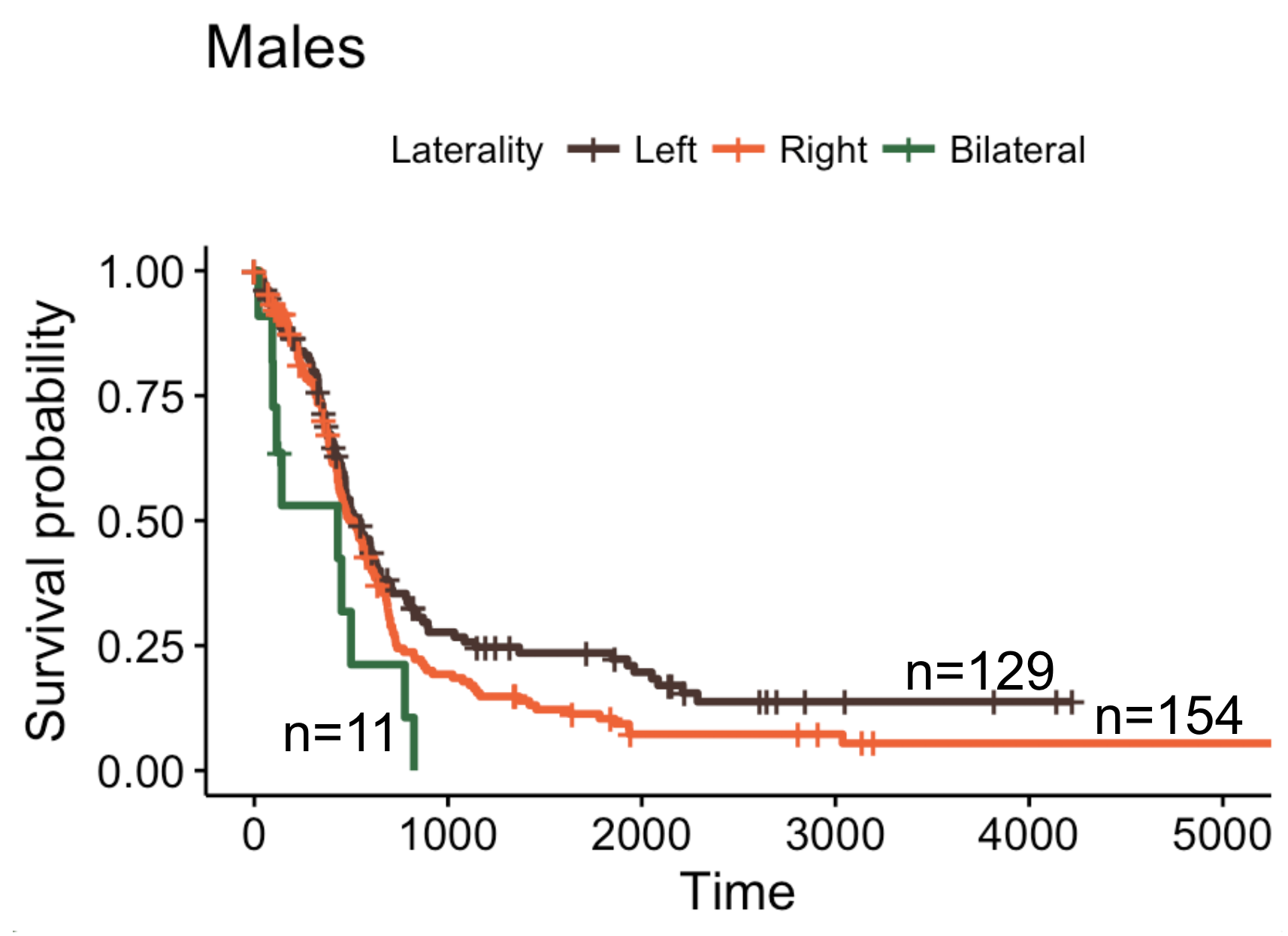

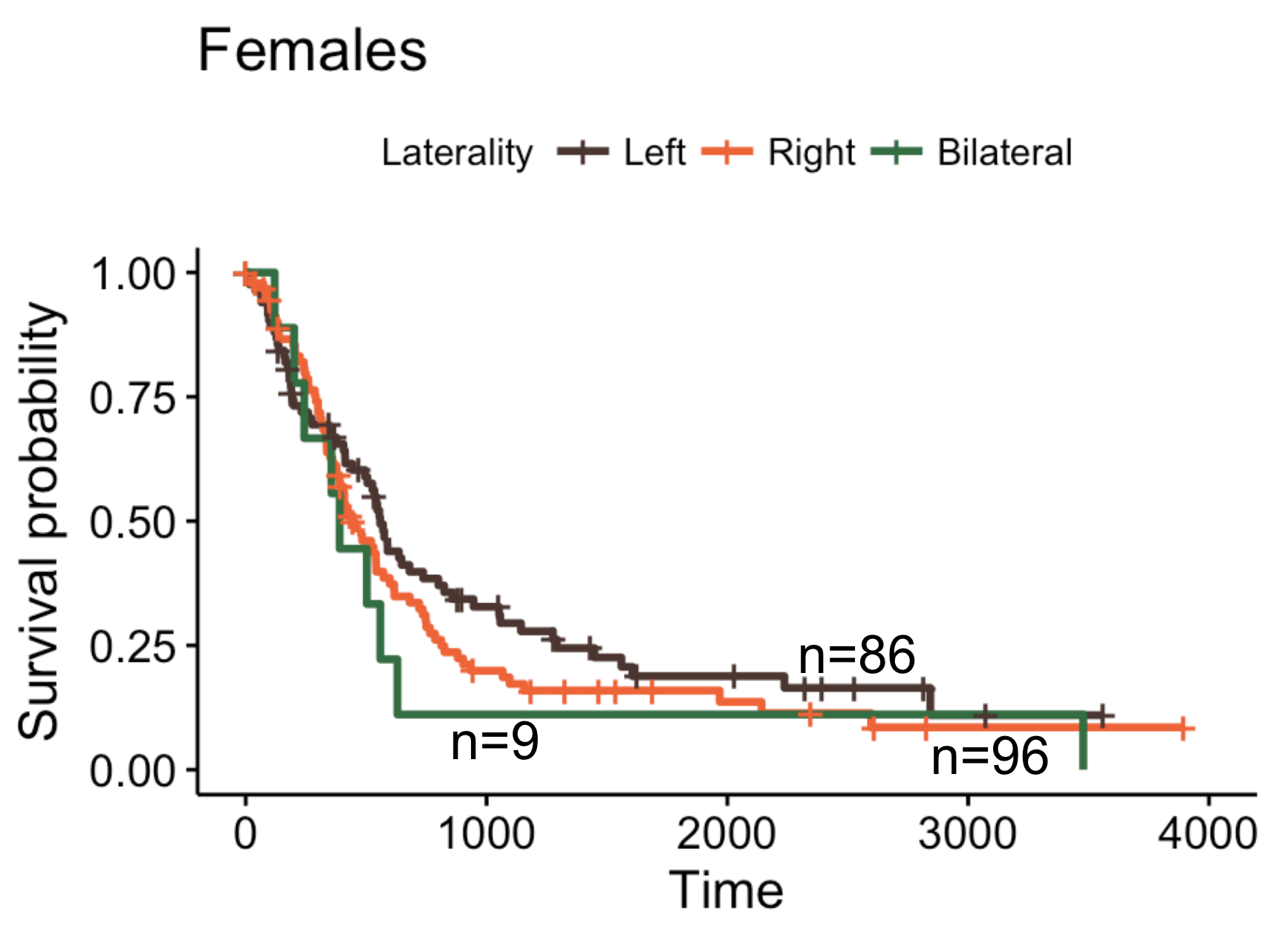


**Supplement 6.** Males with tumors in the left hemisphere trended towards significantly better survival than males with tumors in the right hemisphere (p=0.077) and had significantly better overall survival than males with bilateral tumors (p=0.010). There was no significant difference in female survival when comparing patients with left hemisphere, right hemispheres, and bilateral tumors (right vs left p=0.218, right vs bilateral p=0.471, left vs bilateral p=0.272).


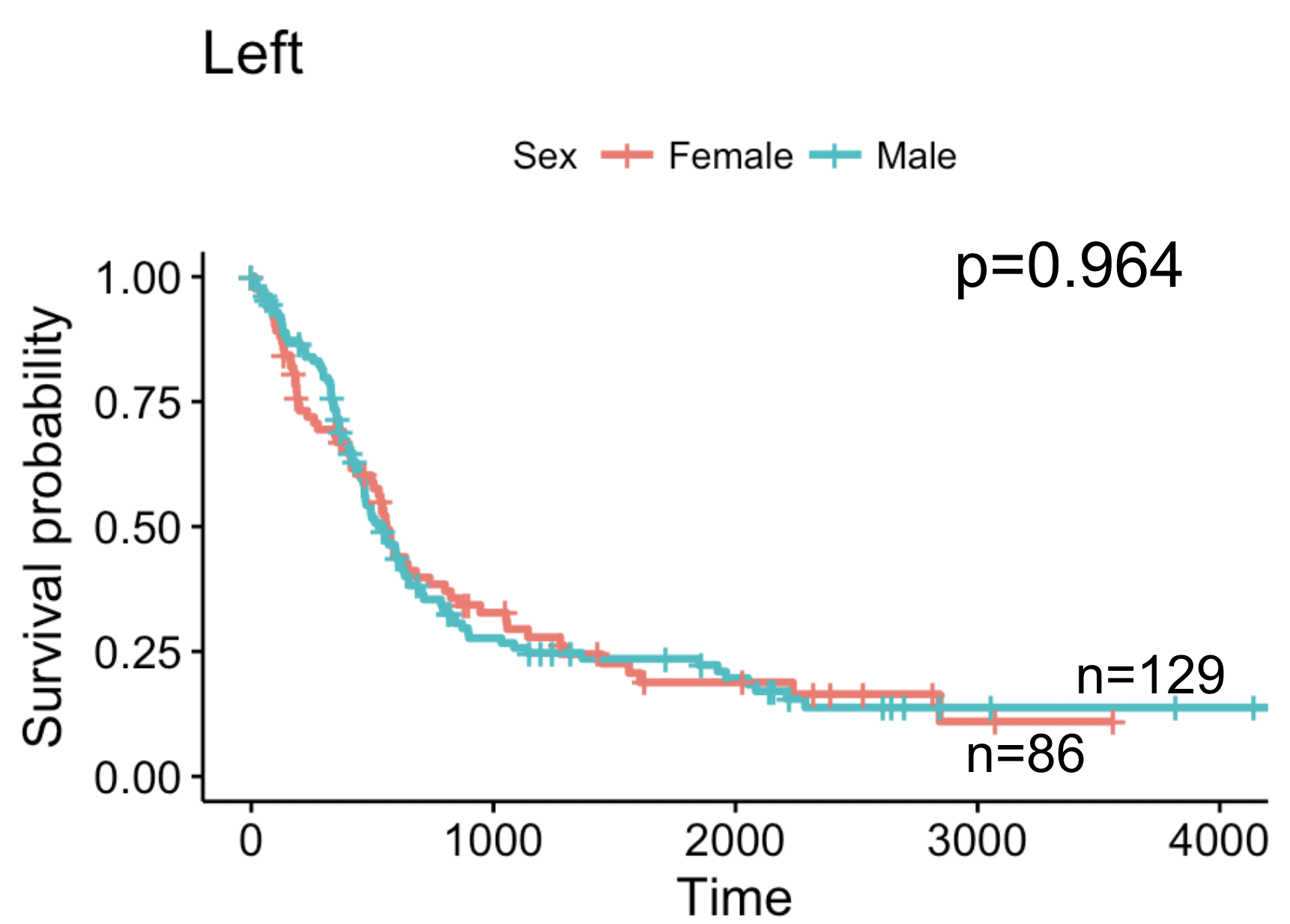

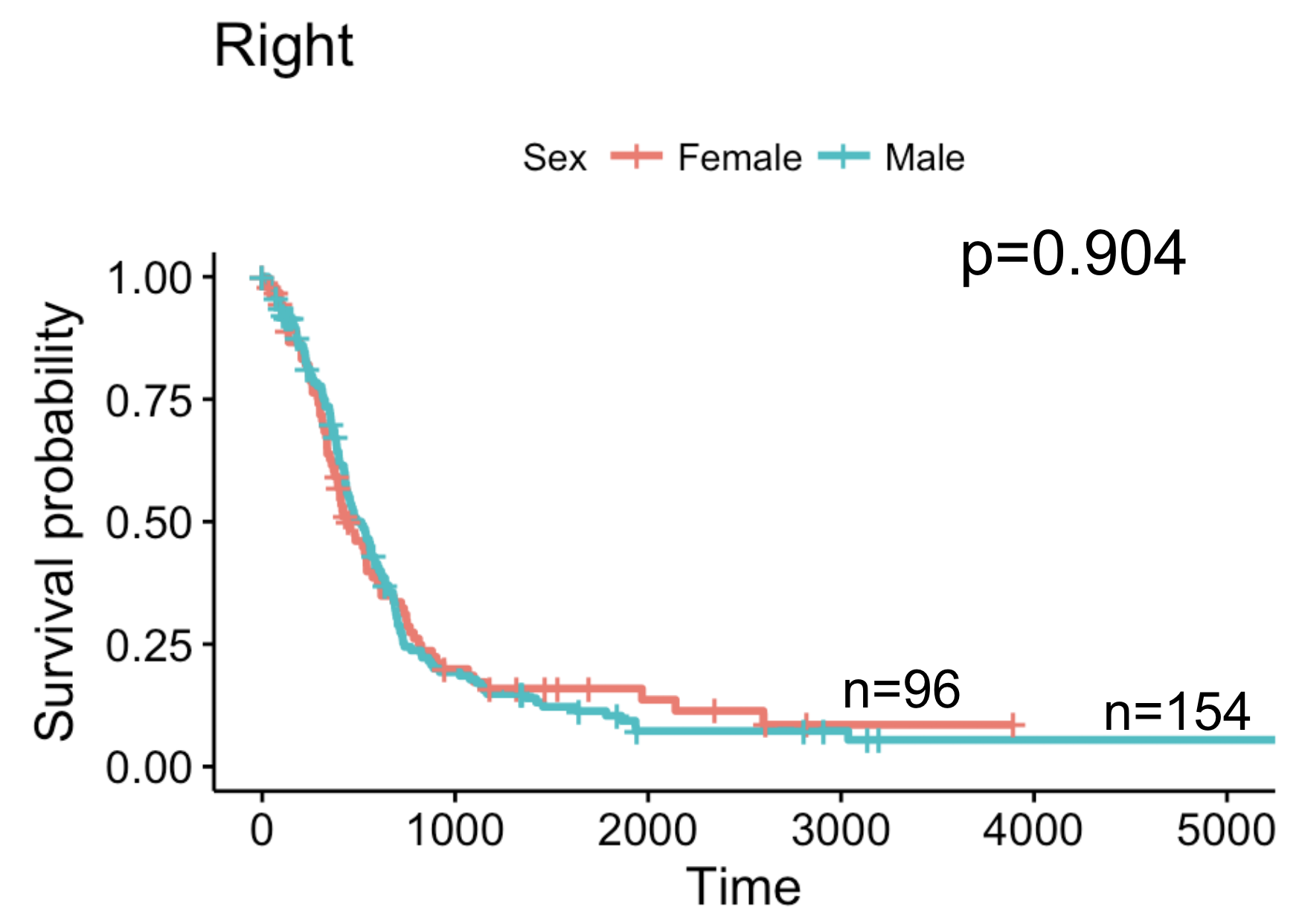

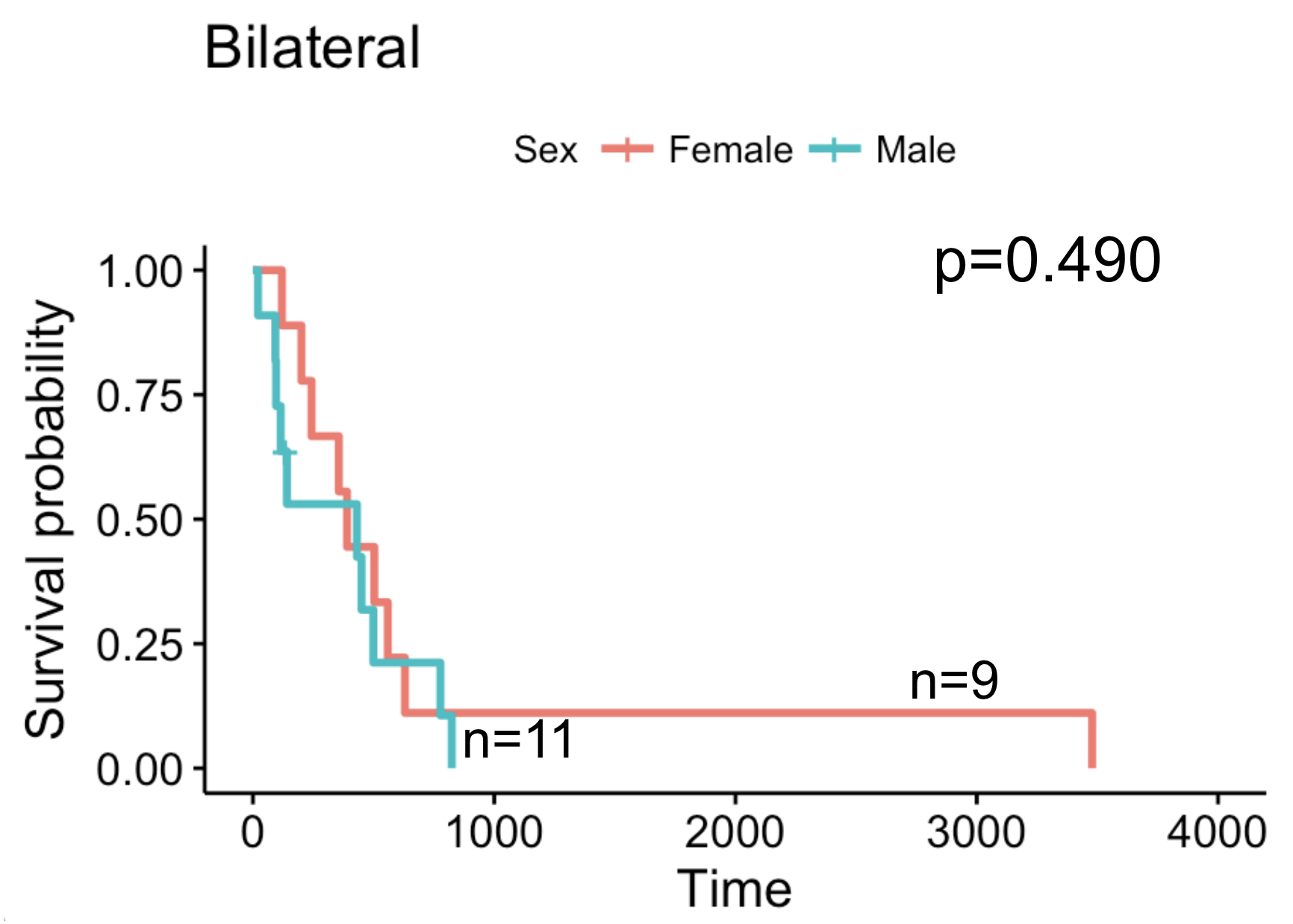


**Supplement 7.** No difference in survival between males and females among left, right, and bilaterally located tumors.

**Extent of Resection**


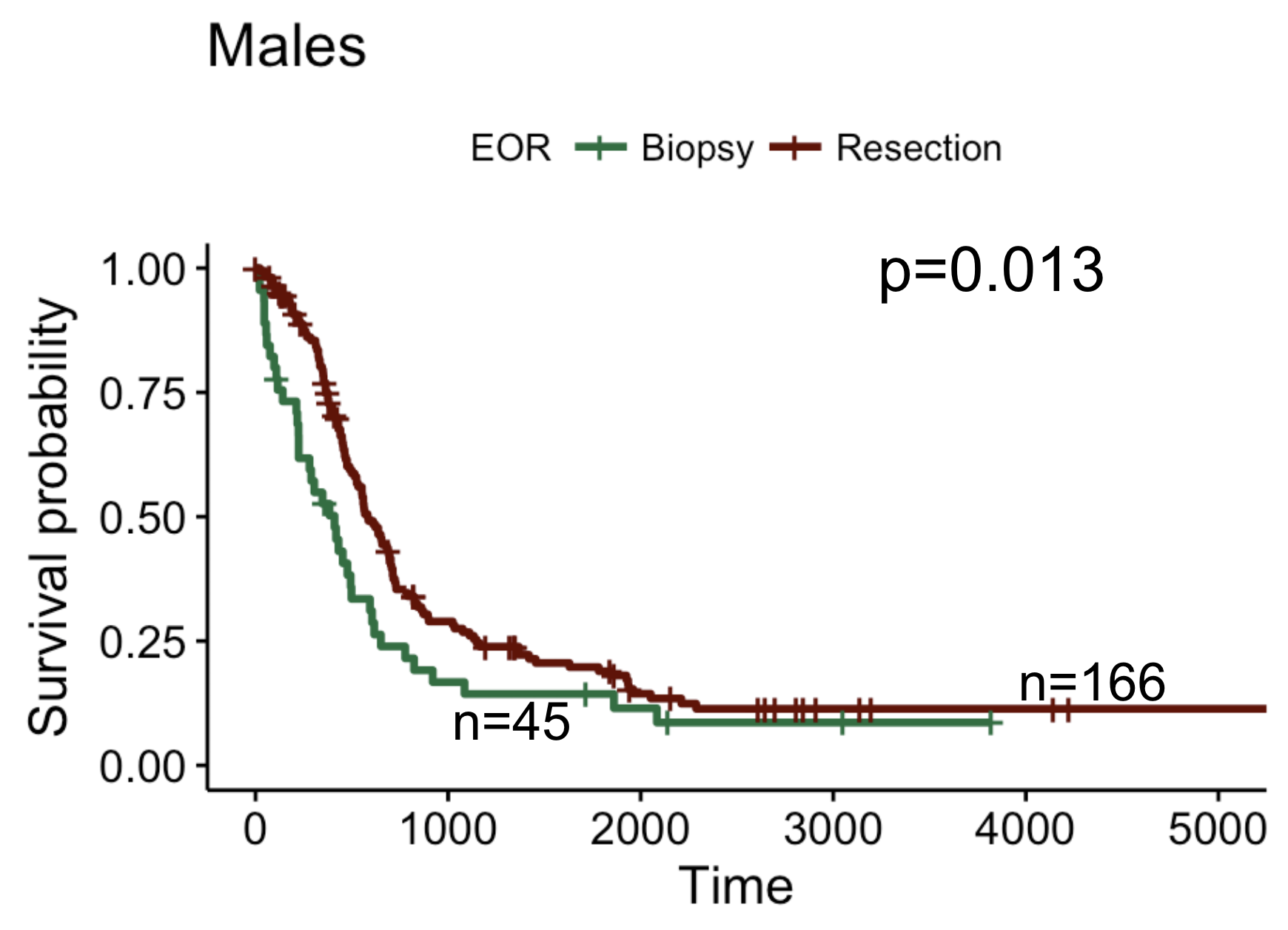

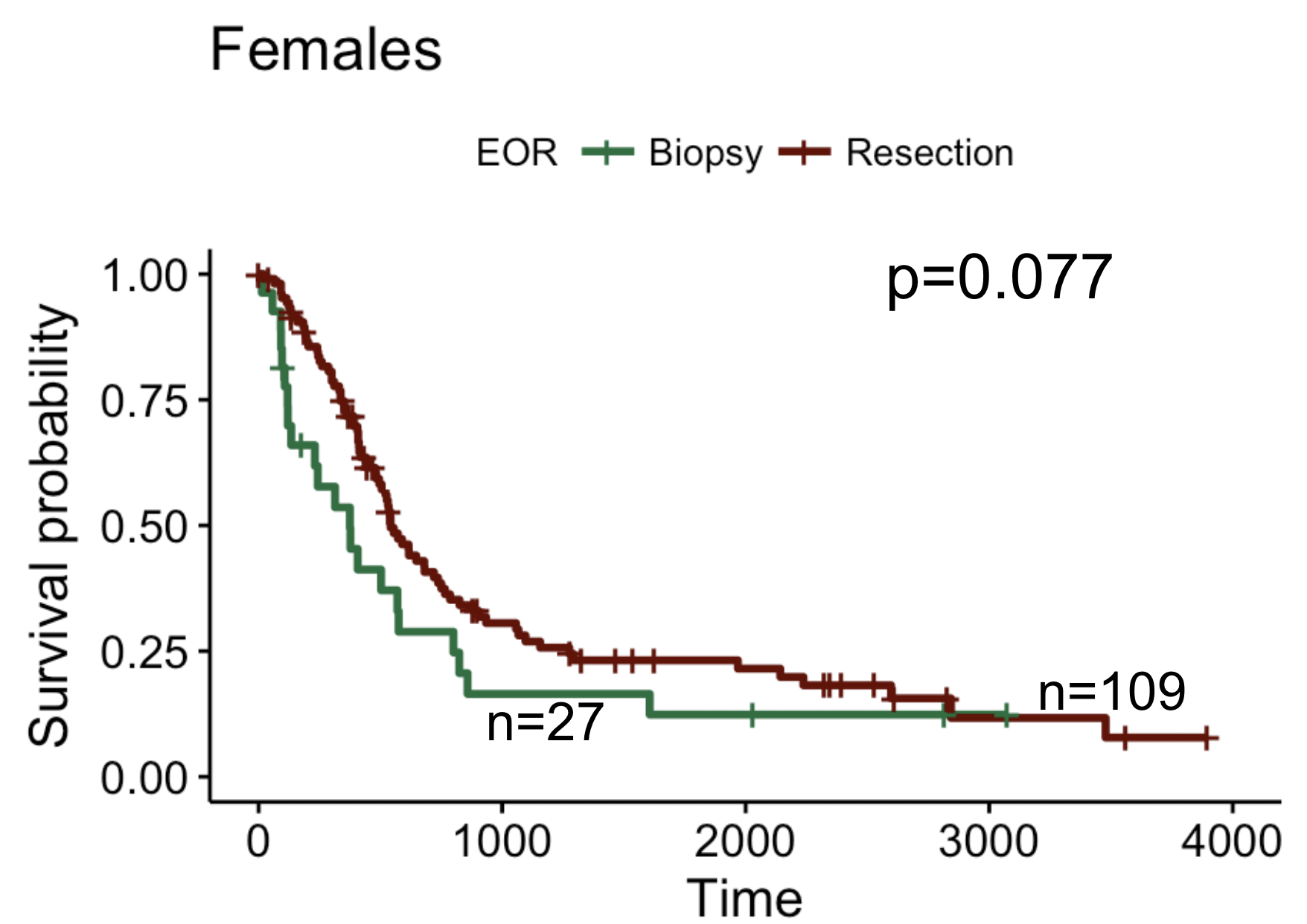


**Supplement 8.** Both males and females showed improved survival when they received some resection (GTR or STR) compared to just receiving a biopsy.


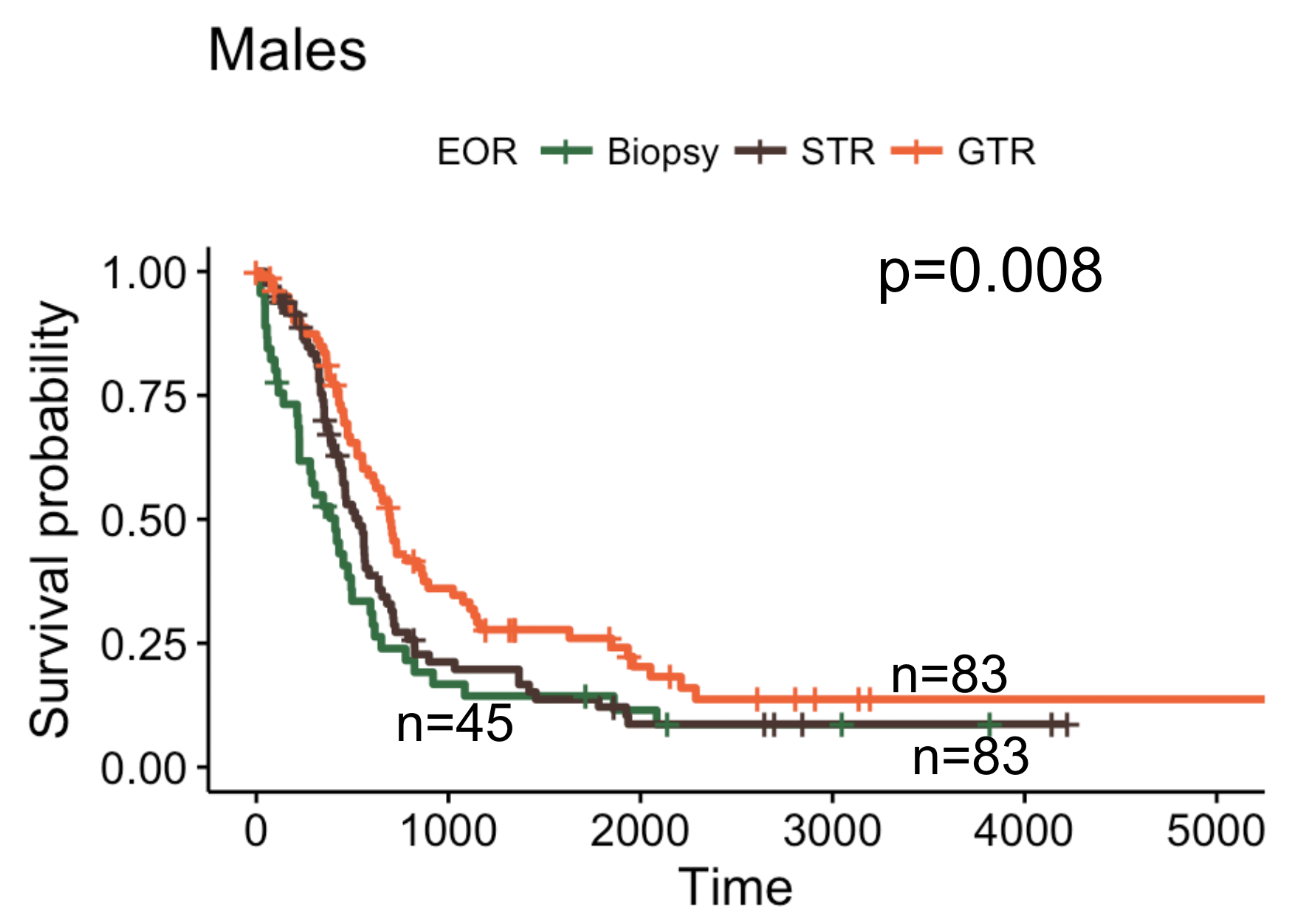

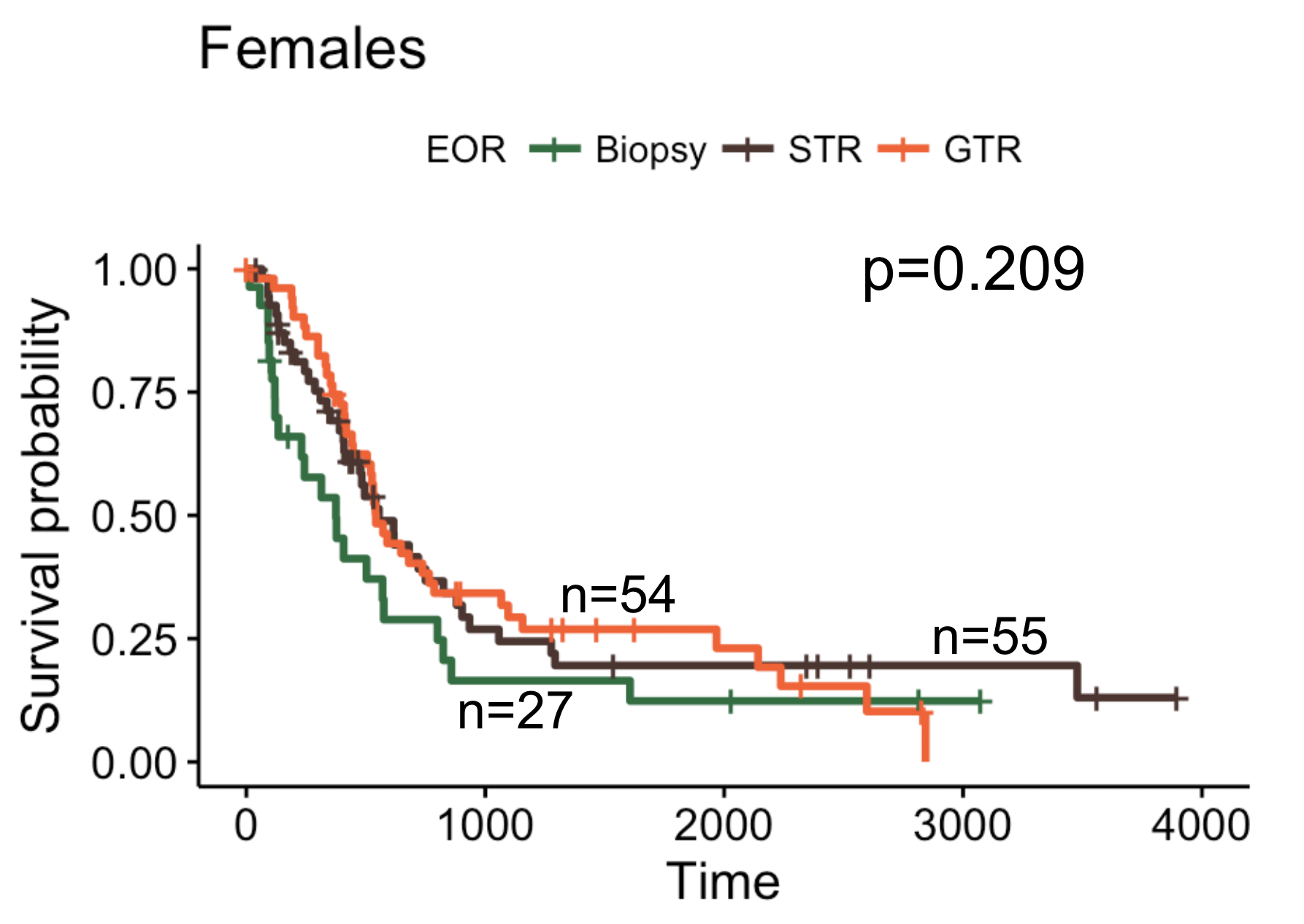


**Supplement 9.** Males have a significant trend of improved survival when patients who received the three kinds of resection (biopsy, STR, and GTR) are compared. This trend is not significant among females. Males who received GTR had significantly better survival than males who received STR (p=0.033). This comparison was not significant among females (p=0.992).


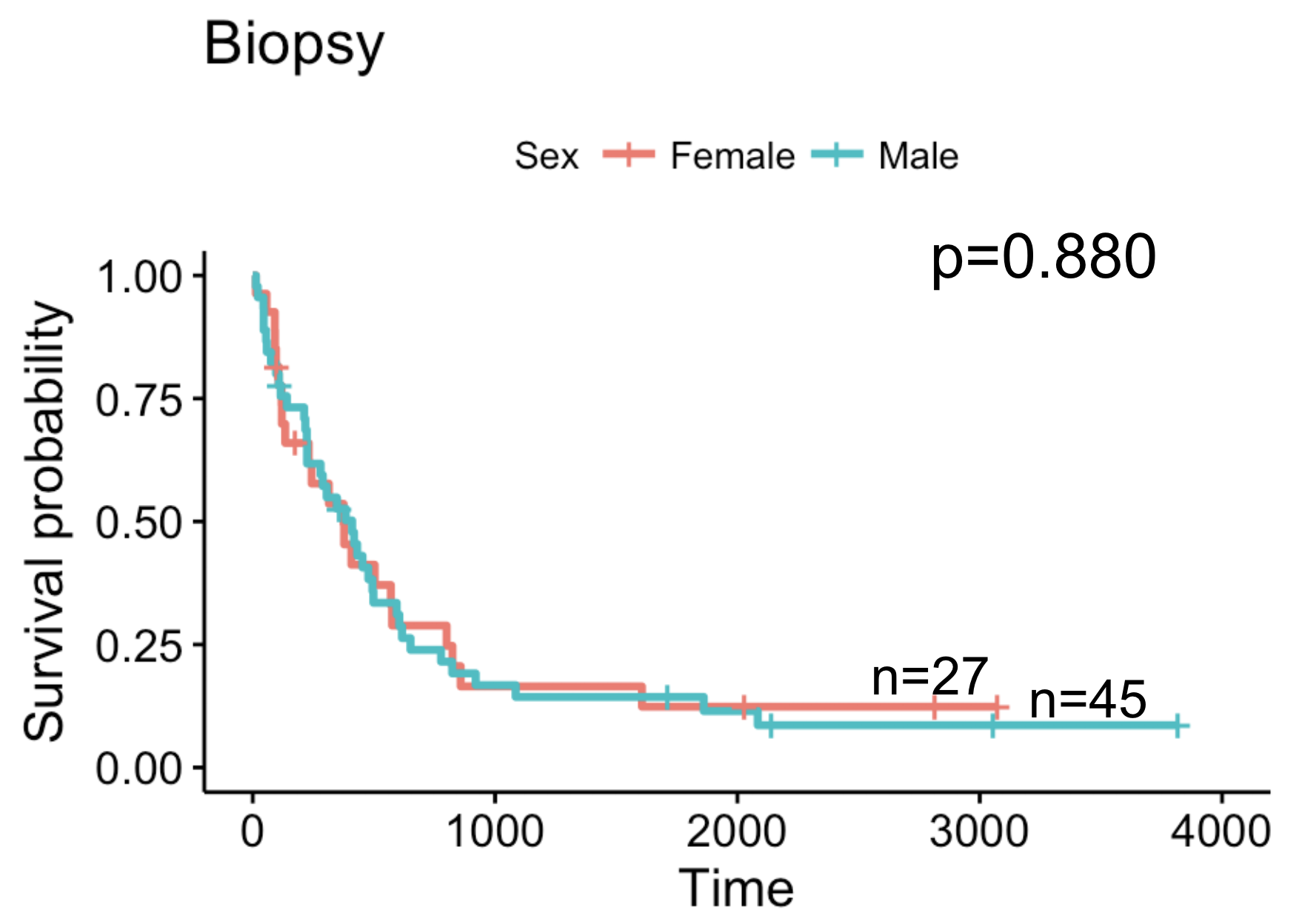

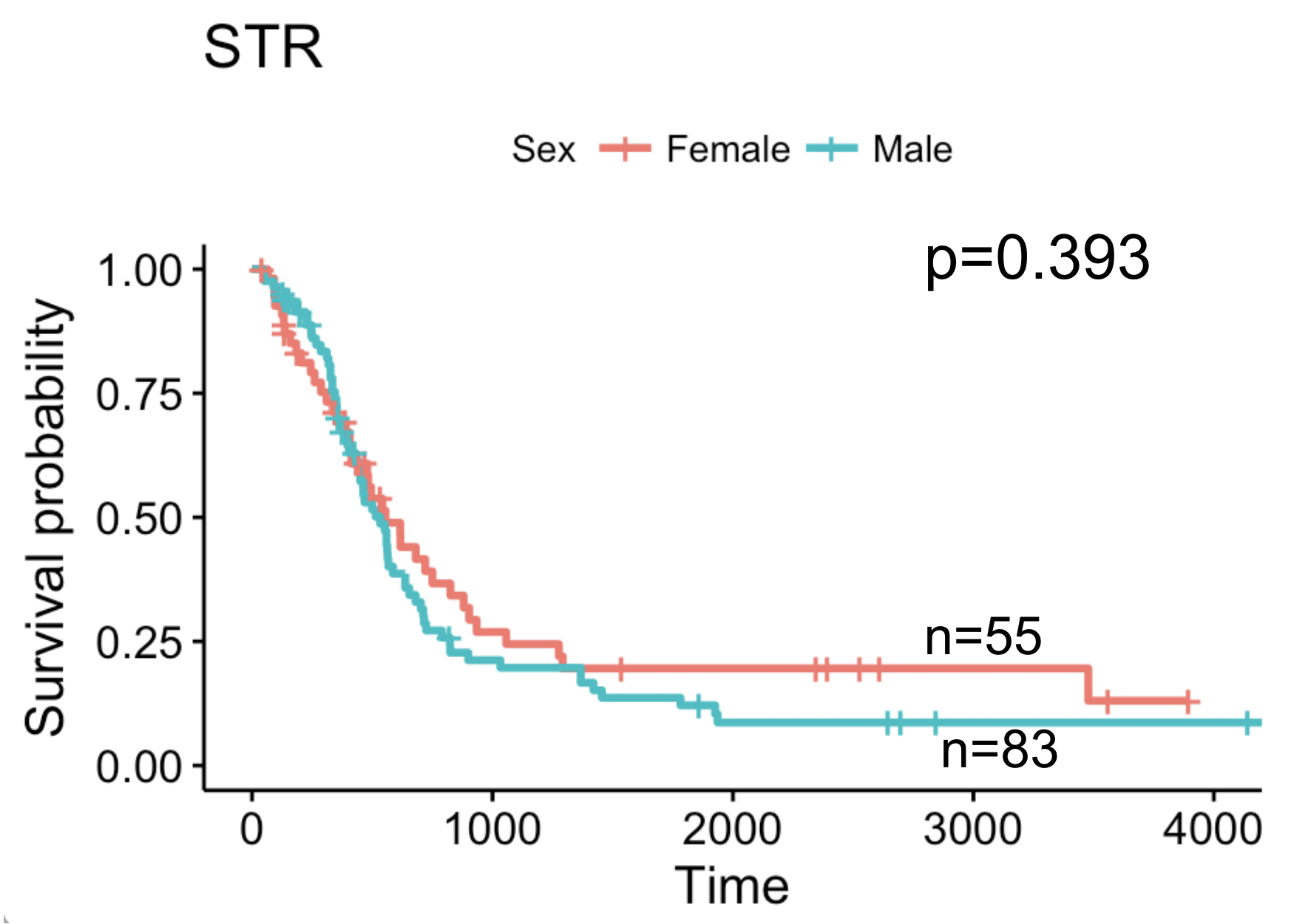

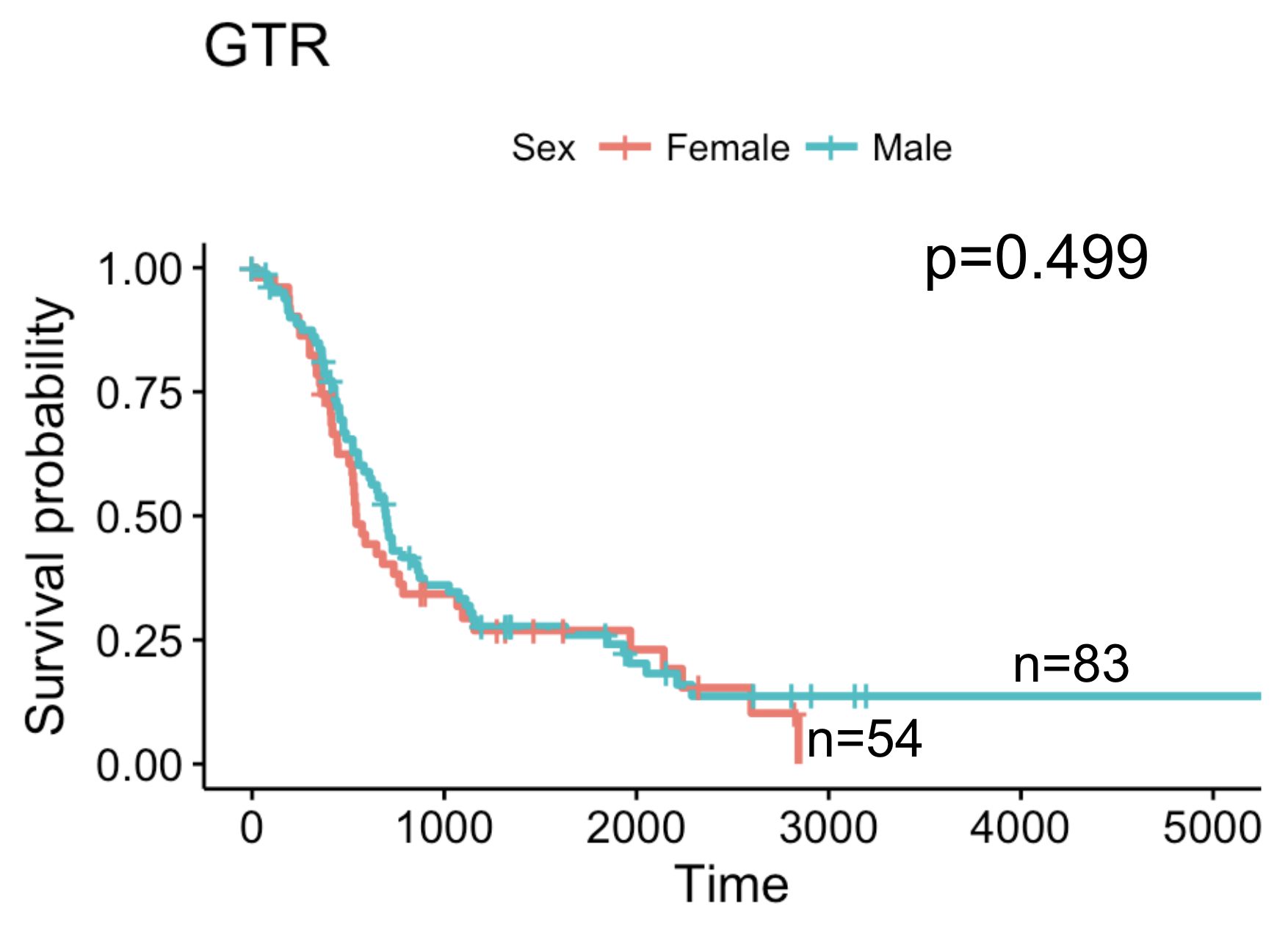


**Supplement 10.** No difference in survival between males and females among biopsy, STR, and GTR patients.

| Covariate | M v F t-test | M v F KS | M v F M-W |
| --- | --- | --- | --- |
| Age | 0.546 | 0.122 | 0.220 |
| Necrosis radius | 0.979 | 0.723 | 0.902 |
| T1Gd radius | 0.657 | 0.254 | 0.618 |
| CE Thickness | 0.365 | 0.452 | 0.226 |
| T2/FLAIR radius | 0.870 | 0.912 | 0.824 |
| Necrosis radius / T1Gd radius | 0.906 | 0.408 | 0.870 |
| CE Thickness / T1Gd radius | 0.977 | 0.370 | 0.820 |
| Necrosis radius / (T2/FLAIR radius) | 0.602 | 0.735 | 0.809 |
| CE Thickness / (T2/FLAIR radius) | 0.719 | 0.243 | 0.696 |
| T1Gd radius / (T2/FLAIR radius) | 0.777 | 0.992 | 0.929 |
| PIHNA D | 0.282 | 0.594 | 0.598 |
| PIHNA ϱ | 0.502 | 0.967 | 0.719 |
| PI D/ϱ | 0.742 | 0.108 | 0.303 |

**Supplement 11:** Table of p-values for the following tests for males vs females: t-test with Welch’s correction, Kolmogorov-Smirnov distribution test, and Mann-Whitney distribution test (n=494)

| Covariate | EXS M vs EXS F t-test | STS M vs STS F t-test |
| --- | --- | --- |
| Age | 0.559 | 0.981 |
| Necrosis radius | 0.203 | 0.464 |
| T1Gd radius | 0.061 | 0.333 |
| CE Thickness | 0.387 | 0.609 |
| T2/FLAIR radius | 0.544 | 0.881 |
| Necrosis radius / T1Gd radius | 0.443 | 0.655 |
| CE Thickness / T1Gd radius | 0.443 | 0.655 |
| Necrosis radius / (T2/FLAIR radius) | 0.467 | 0.656 |
| CE Thickness / (T2/FLAIR radius) | 0.514 | 0.993 |
| T1Gd radius / (T2/FLAIR radius) | 0.203 | 0.620 |
| PIHNA D | 0.166 | 0.285 |
| PIHNA ϱ | **0.023** | 0.787 |
| PI D/ϱ | 0.600 | 0.495 |

**Supplement 12:** Table of p-values for t-test with Welch’s corrections comparing EXS males vs EXS females and STS males vs STS females (N=135)

| **Males** | Mean∓SD | | | | t-Test p-values | | |
| --- | --- | --- | --- | --- | --- | --- | --- |
| **Covariate** | EXS | Non-EXS | STS | Non-STS | EXS vs Non-EXS | EXS vs STS | STS vs Non-STS |
| Age | 51.33 ∓ 11.92 | 58.28 ∓ 13.62 | 65.33 ∓ 15.08 | 56.17 ∓ 12.86 | **0.005** | **<0.001** | **<0.001** |
| Necrosis radius | 11.02 ∓ 5.31 | 11.39 ∓ 5.80 | 12.44 ∓ 5.54 | 11.15 ∓ 5.86 | 0.722 | 0.267 | 0.157 |
| T1Gd radius | 18.79 ∓ 5.41 | 19.53 ∓ 6.08 | 21.49 ∓ 5.67 | 19.09 ∓ 6.01 | 0.485 | **0.041** | **0.011** |
| CE Thickness | 7.771 ∓ 2.89 | 8.177 ∓ 3.10 | 9.052 ∓ 3.06 | 7.970 ∓ 3.05 | 0.472 | 0.069 | **0.031** |
| T2/FLAIR radius | 27.82 ∓ 5.89 | 27.03 ∓ 6.68 | 28.15 ∓ 5.44 | 26.91 ∓ 6.79 | 0.572 | 0.839 | 0.266 |
| PIHNA D | 32.29 ∓ 25.35 | 32.34 ∓ 22.60 | 43.54 ∓ 28.30 | 30.46 ∓ 21.32 | 0.992 | 0.124 | **0.017** |
| PIHNA ϱ | 25.34 ∓ 25.10 | 70.77 ∓ 206.80 | 115.3 ∓ 244.89 | 57.61 ∓ 186.02 | **0.004** | **0.047** | 0.211 |
| PI D/ϱ | 2.636 ∓ 3.04 | 2.051 ∓ 2.02 | 2.061 ∓ 1.81 | 2.126 ∓ 2.22 | 0.330 | 0.380 | 0.846 |

| **Females** | Mean ∓SD | | | | T-Test p-values | | |
| --- | --- | --- | --- | --- | --- | --- | --- |
| **Covariate** | EXS | Non-EXS | STS | Non-STS | EXS vs Non-EXS | EXS vs STS | STS vs Non-STS |
| Age | 48.29 ∓ 19.11 | 59.36 ∓ 14.08 | 65.26 ∓ 10.29 | 56.47 ∓ 15.38 | **0.032** | **0.003** | **<0.001** |
| Necrosis radius | 8.691 ∓ 6.19 | 11.62 ∓ 6.27 | 11.51 ∓ 6.18 | 11.32 ∓ 6.36 | 0.079 | 0.123 | 0.857 |
| T1Gd radius | 15.56 ∓ 5.52 | 19.58 ∓ 6.40 | 20.17 ∓ 6.87 | 18.96 ∓ 6.28 | **0.010** | **0.010** | 0.307 |
| CE Thickness | 6.867 ∓ 3.64 | 7.963 ∓ 3.31 | 8.661 ∓ 3.98 | 7.647 ∓ 3.13 | 0.248 | 0.105 | 0.132 |
| T2/FLAIR radius | 25.91 ∓ 9.27 | 27.09 ∓ 6.80 | 28.38 ∓ 6.36 | 26.61 ∓ 7.17 | 0.688 | 0.432 | 0.226 |
| PIHNA D | 19.7 ∓ 17.24 | 37.92 ∓ 40.07 | 59.52 ∓ 62.33 | 30.34 ∓ 27.21 | **0.008** | **0.003** | **0.018** |
| PIHNA ϱ | 10.79 ∓ 9.46 | 87.89 ∓ 254.54 | 105.0 ∓ 221.44 | 75.73 ∓ 251.98 | **0.001** | **0.027** | 0.535 |
| PI D/ϱ | 3.39 ∓ 4.82 | 1.998 ∓ 2.02 | 2.432 ∓ 2.63 | 2.035 ∓ 2.34 | 0.303 | 0.493 | 0.427 |

**Supplement 13:** Full table of t-test results

| **Males** | **Univariate** | | | **Multivariate** | | |
| --- | --- | --- | --- | --- | --- | --- |
| Covariate | HR | 95% CI | p-value | HR | 95% CI | p-value |
| Age | 1.027 | 1.018-1.037 | **<0.001** | 1.020 | 0.999-1.040 | 0.051 |
| Necrosis radius | 1.018 | 0.996-1.040 | 0.118 |  |  | N/A |
| T1Gd radius | 1.024 | 1.003-1.046 | **0.025** | 0.967 | 0.909-1.029 | 0.288 |
| CE Thickness | 1.028 | 0.989-1.068 | 0.161 |  |  | N/A |
| T2/FLAIR radius | 0.996 | 0.972-1.020 | 0.744 |  |  | N/A |
| Necrosis radius / T1Gd radius | 1.089 | 0.584-2.028 | 0.789 |  |  | N/A |
| CE Thickness / T1Gd radius | 0.910 | 0.483-1.714 | 0.771 |  |  | N/A |
| Necrosis radius / (T2/FLAIR radius) | 1.787 | 0.740-4.318 | 0.197 |  |  | N/A |
| CE Thickness / (T2/FLAIR radius) | 1.897 | 0.689-5.222 | 0.215 |  |  | N/A |
| T1Gd radius / (T2/FLAIR radius) | 3.278 | 1.267-8.481 | **0.014** | 7.485 | 0.334-167.7 | 0.205 |
| PIHNA D | 1.003 | 0.997-1.010 | 0.266 |  |  | N/A |
| PIHNA ϱ | 1.001 | 1.000-1.001 | 0.064 | 1.000 | 0.999-1.001 | 0.942 |
| PI D/ϱ | 0.932 | 0.872-0.996 | **0.038** | 1.006 | 0.852-1.187 | 0.948 |
| IDH1 Mutation | 1.021 | 0.461-2.259 | 0.959 |  |  | N/A |
| MGMT Methylation | 0.412 | 0.201-0.845 | **0.016** | 0.342 | 0.148-0.791 | **0.012** |
| EOR | 0.732 | 0.601-0.891 | **0.002** | 0.831 | 0.598-1.155 | 0.271 |
| Laterality | 1.271 | 0.974-1.659 | 0.077 | 1.879 | 1.117-3.159 | **0.017** |

| **Females** | **Univariate** | | | **Multivariate** | | |
| --- | --- | --- | --- | --- | --- | --- |
| Covariate | HR | 95% CI | p-value | HR | 95% CI | p-value |
| Age | 1.028 | 1.015-1.041 | **<0.001** | 1.028 | 1.008-1.049 | **0.006** |
| Necrosis radius | 1.017 | 0.991-1.042 | 0.204 |  |  | N/A |
| T1Gd radius | 1.026 | 1.000-1.052 | **0.048** | 0.988 | 0.952-1.025 | 0.508 |
| CE Thickness | 1.037 | 0.988-1.088 | 0.143 |  |  | N/A |
| T2/FLAIR radius | 1.017 | 0.989-1.045 | 0.232 |  |  | N/A |
| Necrosis radius / T1Gd radius | 1.329 | 0.625-2.825 | 0.460 |  |  | N/A |
| CE Thickness / T1Gd radius | 0.753 | 0.354-1.600 | 0.460 |  |  | N/A |
| Necrosis radius / (T2/FLAIR radius) | 1.139 | 0.458-2.833 | 0.779 |  |  | N/A |
| CE Thickness / (T2/FLAIR radius) | 1.429 | 0.329-6.204 | 0.634 |  |  | N/A |
| T1Gd radius / (T2/FLAIR radius) | 1.326 | 0.539-3.257 | 0.539 |  |  | N/A |
| PIHNA D | 1.011 | 1.006-1.016 | **<0.001** | 1.009 | 1.001-1.017 | **0.031** |
| PIHNA ϱ | 1.001 | 1.000-1.002 | 0.052 | 1.000 | 0.999-1.002 | 0.609 |
| PI D/ϱ | 0.996 | 0.937-1.059 | 0.906 |  |  | N/A |
| IDH1 Mutation |  |  | N/A |  |  | N/A |
| MGMT Methylation | 0.287 | 0.109-0.751 | **0.011** | 0.311 | 0.080-1.211 | 0.092 |
| EOR | 0.833 | 0.639-1.088 | 0.180 | 0.793 | 0.560-1.122 | 0.191 |
| Laterality | 1.238 | 0.882-1.737 | 0.218 | 1.291 | 0.798-2.089 | 0.299 |

**Supplement 14:** Table of extended CPH analysis that includes both categorical and quantitative variables. Variable definitions: Laterality (1 = left, 2 = right), EOR (1=biopsy, 2 = STR, 3 = GTR), IDH1 (0 = wt, 1 = mut), MGMT (1 = unmethylated, 2 = methylated)

**Supplement 15: Analysis on Stupp protocol patients only**

|  | **Stupp Protocol Group**  **N=179** | |
| --- | --- | --- |
|  | **Male** | **Female** |
| **All Patients** | 113 | 66 |
| **Extreme (OS>1825 days)** | 18 | 12 |
| **Short term (OS<210 days)** | 1 | 5 |

**Supplement 15A:** Number of males and females in each survival group

| **Males** | **Univariate** | | | **Multivariate** | | |
| --- | --- | --- | --- | --- | --- | --- |
| Covariate | HR | 95% CI | p-value | HR | 95% CI | p-value |
| Age | 1.006 | 0.989-1.025 | 0.467 |  |  |  |
| Necrosis radius | 1.014 | 0.979-1.050 | 0.447 |  |  |  |
| T1Gd radius | 1.022 | 0.989-1.056 | 0.201 |  |  |  |
| CE Thickness | 1.033 | 0.976-1.095 | 0.264 |  |  |  |
| T2/FLAIR radius | 0.994 | 0.952-1.037 | 0.781 |  |  |  |
| PIHNA D | 0.999 | 0.989-1.010 | 0.852 |  |  |  |
| PIHNA ϱ | 1.003 | 0.999-1.007 | **0.089** | 1.007 | 1.001-1.014 | **0.026** |
| PI D/ϱ | 0.876 | 0.774-0.991 | **0.035** | 0.916 | 0.792-1.059 | 0.235 |

| **Females** | **Univariate** | | | **Multivariate** | | |
| --- | --- | --- | --- | --- | --- | --- |
| Covariate | HR | 95% CI | p-value | HR | 95% CI | p-value |
| Age | 1.024 | 1.003-1.045 | **0.027** | 1.024 | 0.999-1.050 | 0.052 |
| Necrosis radius | 1.007 | 0.965-1.052 | 0.742 |  |  |  |
| T1Gd radius | 1.010 | 0.965-1.058 | 0.672 |  |  |  |
| CE Thickness | 1.008 | 0.916-1.108 | 0.876 |  |  |  |
| T2/FLAIR radius | 1.014 | 0.965-1.065 | 0.581 |  |  |  |
| PIHNA D | 1.013 | 0.999-1.027 | **0.066** | 1.014 | 1.001-1.027 | **0.037** |
| PIHNA ϱ | 1.001 | 0.999-1.003 | 0.137 |  |  |  |
| PI D/ϱ | 0.984 | 0.864-1.121 | 0.811 |  |  |  |

**Supplement 15B:** Results of univariate and multivariate CPH analyses for males and females. Factors that were significant or almost significant (p<0.10) in univariate analysis were included in the multivariate analysis.

**Supplement 16:**

The Proliferation-Invasion (PI) model is based on two key parameters: the net rate of proliferation, ϱ, and the net rate of invasion, D. By assuming that the T1Gd abnormality coincides with one threshold of tumor cell density relative to the maximum carrying capacity and the T2/FLAIR abnormality coincides with another, lower threshold, one can obtain patient-specific estimates of these two parameters using standard pre-tx imaging (Swanson KR. Mathematical modeling of the growth and control of tumors: University of Washington; 1999). This calibration uses two sets of MRI data: one set consists of two pre-tx time points of either T1Gd or T2/FLAIR images and the second set consists of one time point with both T1Gd and T2/FLAIR images. The first set is used to calculate a velocity and the second set is used to estimate the degree of invasion.

The PIHNA model similarly relies on an analogous net rate of invasion and net rate of proliferation and does not require two pretreatment image time points. In the PIHNA calibration approach, the relative size of necrosis is used as a surrogate to the velocity estimate from the two time points of imaging. For the calibration method, a lookup table was created, with each entry being an output from a unique D and ρ PIHNA simulation. The lookup table contains the estimated PI D/ρ value, the size of necrosis, the T1Gd visible portion of the tumor (assumed at >80% cell density) and the T2/FLAIR visible portion (assumed at >16% cell density), throughout each simulation. Given a patient’s T1Gd volume, T2/FLAIR volume, necrosis size, and the PI estimated D/ρ at one time point, the lookup table points to a sub­table of simulation points that match the T2/FLAIR size on MRI within a small measurement tolerance. A D and ρ is then chosen based on the simulations that match the PI D/ρ and necrosis size within a small tolerance. If multiple D ϱ pairs exist that satisfy the aforementioned criteria, the pair that minimizes the T1Gd size is chosen.

**Supplement 17:** Tumor volume segmentation and processing

All tumor regions were segmented using our in-house thresholding software. This software is written in python and allows the user to set a threshold, per slice, such that regions are only shown whose intensities are greater than the given threshold. The user can then draw around the area of interest with their mouse and all voxels within the drawn region above the selected threshold will be saved as the tumor region. Multiple trained individuals were utilized to segment the hundreds of images utilized in this study. For quality assurance, all segmentations were subject to review by a qualified technician.

Since the MR images come from a variety of institutions, the MRI scan parameters vary. The vast majority of MR images used in this study are between 256 x 256 and 512 x 512 in matrix size, 1-5 mm in slice thickness, and 1.5-3 Tesla in magnetic strength. The volume of the segmented region is calculated based on the number of voxels in the segmented region and the volume of a voxel per the image. As the model calculations for D and ϱ are based on analysis dependent on a spherically-symmetric radial growth assumption, these volumes are then converted to their spherically-equivalent radial value.
